# Phenotype-specific association of C-reactive protein-to-lymphocyte ratio with incident proteinuric CKD versus isolated eGFR decline: a real-world retrospective cohort study

**DOI:** 10.3389/fendo.2026.1769059

**Published:** 2026-04-01

**Authors:** Wenbo Yang, Wen Jiang, Qing Zhu, Mengru Wang, Heizhati Mulalibieke, Luo Qin, Ting Wu, Bingxuan Guo, Jing Hong, Nanfang Li

**Affiliations:** 1 Graduate School, Xinjiang Medical University, Urumqi, Xinjiang, China; 2 Hypertension Center of People’s Hospital of Xinjiang Uygur Autonomous Region, Xinjiang Hypertension Institute, Urumqi, China; 3 NHC Key Laboratory of Hypertension Clinical Research, Urumqi, China; 4 Key Laboratory of Xinjiang Uygur Autonomous Region "Hypertension Research Laboratory", Urumqi, China; 5 Xinjiang Clinical Medical Research Center for Hypertension (Cardio-Cerebrovascular Diseases), Urumqi, China

**Keywords:** chronic kidney disease, C-reactive protein-to-lymphocyte ratio, hypertension, inflammation, proteinuria, risk stratification

## Abstract

**Background:**

Residual kidney risk persists in hypertensive patients despite guideline-directed blood pressure (BP) management. Whether systemic inflammation is preferentially associated with the development of distinct early chronic kidney disease (CKD) phenotypes remains uncertain.

**Methods:**

This retrospective cohort study included 5,904 hypertensive patients with preserved baseline kidney function. The primary exposure was the C-reactive protein-to-lymphocyte ratio (CLR). Outcomes assessed were incident proteinuric CKD, isolated estimated glomerular filtration rate (eGFR) decline, and any incident CKD. Phenotype-specific associations were evaluated using multivariable cause-specific Cox models. We additionally assessed the additive interaction between CLR and BP control, and evaluated the incremental predictive value of CLR beyond traditional risk factors.

**Results:**

During a median follow-up of 34.1 months, 598 participants developed proteinuric CKD, 89 developed isolated eGFR decline, and 728 developed any incident CKD. Higher CLR was independently associated with proteinuric CKD (per 1-SD: HR 1.14, 95% CI 1.05–1.24; highest vs lowest quartile: HR 1.46, 95% CI 1.15–1.85) and any incident CKD (per 1-SD: HR 1.13, 95% CI 1.05–1.22), but not with isolated eGFR decline (per 1-SD: HR 1.05, 95% CI 0.85–1.29). A significant additive interaction emerged between high CLR and uncontrolled BP (RERI 0.30), synergistically amplifying renal risk. Adding CLR to traditional risk models significantly improved risk reclassification (NRI 0.083, P = 0.008).

**Conclusions:**

In hypertensive patients, elevated CLR preferentially predicts new-onset proteinuria over isolated eGFR decline. As an accessible biomarker for renal risk stratification, CLR can identify patients requiring vigilant proteinuria surveillance and comprehensive management, particularly those with uncontrolled BP.

## Introduction

Hypertension is a major driver of chronic kidney disease (CKD) globally (1). Despite guideline-directed blood pressure (BP) control and rigorous metabolic management, many patients still develop new-onset albuminuria or experience progressive loss of kidney function (2, 3), highlighting persistent residual renal risk beyond traditional hemodynamic and metabolic factors (4). Identifying pathways associated with this residual risk is critical for more precision risk stratification and prevention in hypertensive populations.

Growing evidence supports low-grade systemic inflammation as a key contributor to target-organ damage, operating largely independently of BP and metabolic factors (5–8). The C-reactive protein-to-lymphocyte ratio (CLR) has emerged as a practical surrogate for this systemic inflammatory burden. By integrating elevated C-reactive protein (reflecting innate immune activation) with lymphopenia (reflecting impaired adaptive immune response under chronic stress) (9–11), CLR reliably captures chronic immunological dysregulation. Although CLR exhibits relevance in various cardiometabolic conditions (12, 13), longitudinal evidence linking it specifically to hypertensive kidney outcomes remains remarkably scarce (14–16).

Crucially, prior epidemiological studies investigating the inflammation-kidney axis have predominantly relied on composite renal endpoints (16, 17). This approach overlooks the phenotypic heterogeneity of hypertensive kidney injury, which clinically diverges into two distinct trajectories: proteinuric CKD and isolated eGFR decline (18–20). Pathophysiologically, incident proteinuria is closely linked to active microvascular endothelial barrier dysfunction (21), whereas isolated eGFR decline often reflects cumulative, hemodynamically driven ischemic sclerosis (22). It remains unelucidated whether systemic inflammation preferentially drives the proteinuric phenotype (23). Distinguishing between these trajectories is of paramount clinical importance, as they represent distinct pathophysiological mechanisms and different prognostic implications. Exploring whether baseline CLR differentially predicts these phenotypes may provide valuable insights into the specific role of inflammation, thereby enabling more precise risk stratification and tailored preventative surveillance.

To address these gaps, we leveraged a large, real-world electronic health record (EHR)-derived retrospective cohort of hypertensive patients with preserved kidney function. To our knowledge, this is the first longitudinal study to explicitly evaluate the phenotype-specific predictive value of CLR. Specifically, we aimed to test whether CLR is differentially associated with incident proteinuric CKD versus isolated eGFR decline, to evaluate the synergistic interaction between this inflammatory burden and BP control, and to quantify its incremental prognostic value beyond traditional clinical risk factors, while benchmarking its performance against other inflammation indices.

## Materials and methods

### Study design and cohort inception

We conducted a single-center, EHR derived, real-world retrospective cohort study, utilizing data from the Hypertension DATAbase at Urumchi (UHDATA) (24). To ensure rigorous outcome ascertainment, the study domain was restricted to patients undergoing standardized inpatient evaluations at the Hypertension Center, hereby defined as the “Standardized Baseline (T0).” The protocol complied with the Declaration of Helsinki, with ethical approval granted by the institutional review board. Given the retrospective design and data anonymization, the requirement for informed consent was waived.

**Figure 1:**
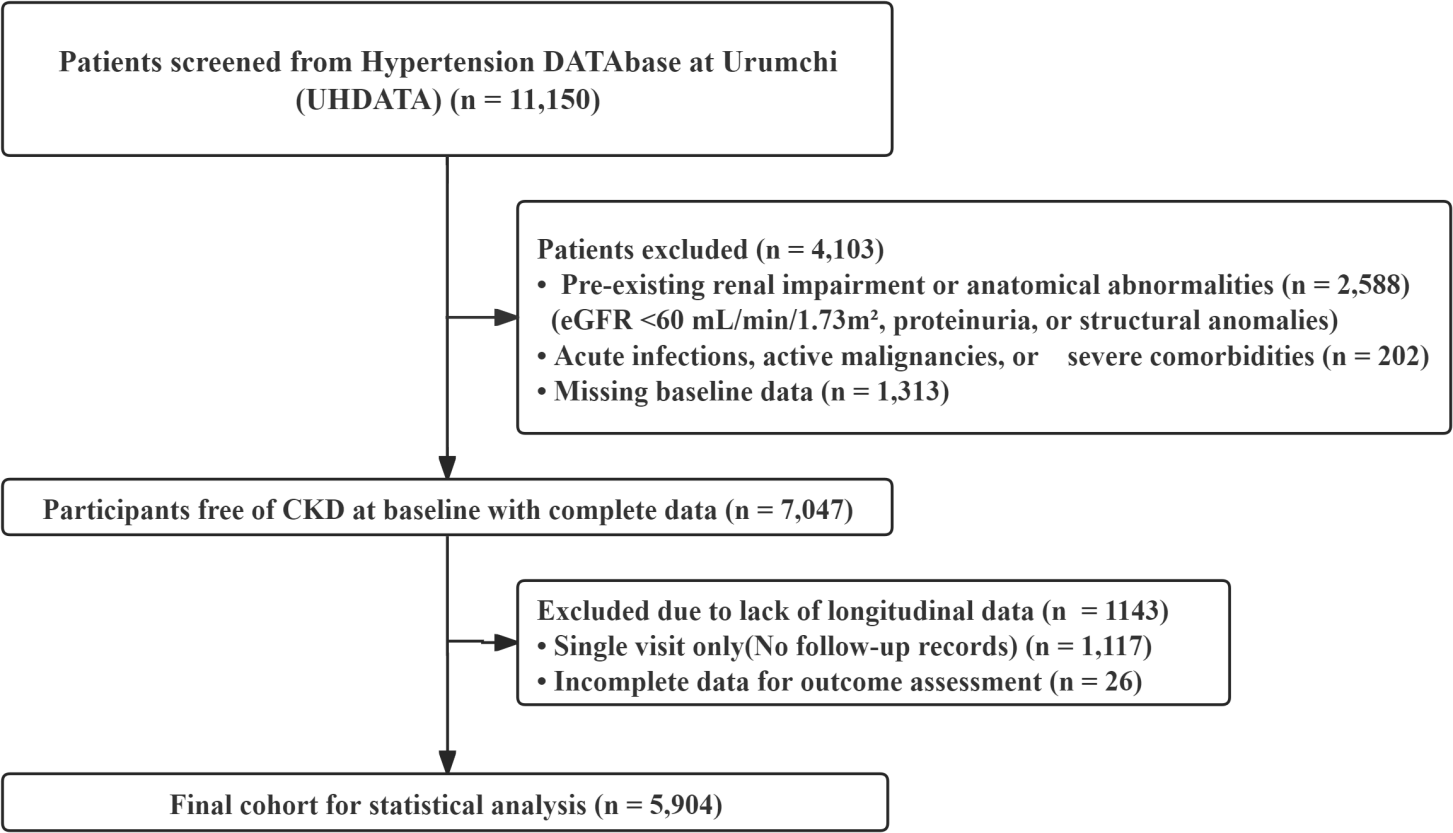
Flow diagram of participant screening and enrollment.

Participants included adults hypertensive patients admitted between January 1, 2015, and June 30, 2023. A dual-validation approach was employed for initial screening: subjects were required to possess (1) a comprehensive baseline evaluation at the Hypertension Center and (2) at least two inpatient admission records to enable rigorous longitudinal outcome assessment and survival analysis. From an initial screen of 11150 individuals, the final analytical cohort (N = 5904) was established after applying strict exclusion criteria (detailed in **Supplementary Methods**). Specifically, we excluded individuals with pre-existing renal impairment (baseline eGFR < 60 mL/min/1.73 m² or prevalent proteinuria), ensuring the cohort was “at risk” for incident events. To isolate the impact of low-grade inflammation, we further excluded participants with confounding inflammatory etiologies (e.g., acute infections, active malignancy, autoimmune disorders) or competing anatomical renal abnormalities (**Figure 1**). Notably, participants for whom the standardized baseline coincided with their last available medical record were excluded to ensure valid longitudinal observation, as these individuals contributed zero person-time to the survival analysis.

### Outcome ascertainment

The primary endpoint was incident proteinuric CKD, operationalized as the first laboratory-confirmed occurrence of proteinuria (defined by any of the following criteria: ACR ≥ 30 mg/g, 24-hour urine protein ≥ 150 mg, or automated semi-quantitative protein ≥ 2+) with a concurrently preserved eGFR (≥ 60 mL/min/1.73 m²). The thresholds for proteinuria were aligned with the established KDIGO criteria for kidney damage (25). For semi-quantitative assessments, we opted for a conservative threshold (≥ 2+) to prioritize diagnostic specificity, effectively mitigating the false-positive misclassifications in large-scale EHR data. Secondary endpoints comprised: (1) isolated eGFR decline, defined as the first laboratory-confirmed occurrence of eGFR < 60 mL/min/1.73 m² in the absence of concurrent proteinuria; and (2) any incident CKD, serving as a comprehensive outcome encompassing incident proteinuric CKD, isolated eGFR decline, and mixed phenotypes presenting simultaneously. In all time-to-event analyses, follow-up person-time was calculated from the standardized baseline to the first laboratory-confirmed occurrence of the respective endpoint. For event-free individuals, follow-up was strictly right-censored at the date of their last available inpatient encounter with valid laboratory data prior to the administrative database lock (December 31, 2023).

### Assessment of exposure and covariates

The primary exposure, the C-reactive protein-to-lymphocyte ratio (CLR), was calculated as serum CRP (mg/L) divided by the lymphocyte count (10^9^/L). To benchmark its predictive performance for incident renal outcomes, six concurrent inflammatory indices (e.g., NLR, SII) were derived (formulations in **Supplementary Methods**). Clinical covariates—including demographics, anthropometrics, lifestyle factors, and medication history—were ascertained at T0 using standardized protocols. Biochemical assays were conducted at the central clinical laboratory using automated enzymatic methods; blood pressure was recorded following a minimum 5-minute rest period.

### Statistical analysis

Analyses were performed using R software (version 4.4.1). Missing data (< 12%) were addressed using multiple imputation by chained equations (MICE). To comprehensively evaluate the primary exposure, CLR was modeled as both a continuous and a categorical variable. For continuous analyses, raw CLR values were natural log-transformed (ln-CLR) and Z-standardized to mitigate right-skewness and facilitate effect size interpretation (detailed in **Supplementary Methods**). For categorical analyses, participants were directly stratified into four groups based on their raw CLR quartiles: Q1 (<0.588), Q2 (≥0.588 to <1.058), Q3 (≥1.058 to <1.815), and Q4 (≥1.815) for consistency.

### Survival modeling and phenotype specificity

Cumulative incidence rates were visualized using Kaplan-Meier estimates and compared via log-rank tests; absolute risk differences (ARD) were also calculated. Multivariable Cox proportional hazards models were employed to estimate hazard ratios (HRs) and 95% CIs. A hierarchical adjustment strategy was implemented: Model 1 adjusted for age, sex, and ethnicity; Model 2 further adjusted for BMI, hypertension duration, BP, lifestyle, and comorbidities; and Model 3 (Fully adjusted) additionally controlled for eGFR, mild baseline proteinuria, metabolic profiles, and medication use. Restricted cubic splines (RCS) were used to examine non-linearity.

To address the phenotypic heterogeneity of incident renal injury, we utilized cause-specific Cox proportional hazards models. In these analyses, when evaluating a specific individual outcome (e.g., incident proteinuric CKD), the occurrence of the alternative phenotype (i.e., isolated eGFR decline) or a concurrent mixed presentation was right-censored. Conversely, for the outcome of any incident CKD, all three phenotypic manifestations were uniformly treated as incident events. Furthermore, to satisfy the epidemiological events-per-variable (EPV) principle and avoid model overfitting, multivariable adjustments for the rarer “isolated eGFR decline” outcome (n = 89) were parsimoniously restricted to Model 2.

### Interaction and predictive metrics

We evaluated biological interaction on the additive scale between CLR and blood pressure control using the relative excess risk due to interaction (RERI), the attributable proportion (AP), and the synergy index (SI). To comprehensively assess the predictive value of CLR, we first benchmarked its discriminative performance against six concurrent inflammatory indices (NLR, PLR, SII, SIRI, CAR, and AISI). Subsequently, the incremental prognostic value of incorporating CLR into the fully adjusted Model 3 was quantified using Harrell’s C-index, time-dependent AUC, continuous net reclassification improvement (NRI), and integrated discrimination improvement (IDI). Finally, clinical utility was evaluated via decision curve analysis (DCA), with the optimal risk-stratification cutoff identified using the Youden index. (detailed in **Supplementary Methods**).

### Robustness checks

Strict sensitivity analyses were conducted to verify robustness: (1) lag-time analyses excluding events within the first 3 and 6 months to mitigate reverse causality; (2) excluding participants with baseline CRP > 10 mg/L; (3) winsorizing CLR at the 99th percentile; (4) repeating analyses in non-smoking, non-drinking, and non-diabetic subgroups; (5) truncating follow-up at 3 years; and (6) calculating the E-value to quantify the potential impact of unmeasured confounding.

## Results

### Baseline characteristics and metabolic clustering

The final analytical cohort comprised 5,904 participants, who were stratified into four groups based on raw CLR quartiles: Q1 (<0.588), Q2 (≥0.588 to <1.058), Q3 (≥1.058 to <1.815), and Q4 (≥1.815) for consistency. As detailed in **Table 1**, elevated CLR levels signaled a distinct clustering of metabolic and hemodynamic derangements. Compared with the lowest quartile (Q1), participants in the highest quartile (Q4) were significantly older, had higher BMI, and bore a heavier burden of uncontrolled hypertension (69.6% vs. 61.1%), hyperuricemia, and dyslipidemia (all P < 0.001). Notably, while baseline renal function (eGFR) appeared preserved and comparable across groups, signs of subclinical structural injury were already evident: the prevalence of microalbuminuria (ACR 10–29.9 mg/g) paralleled the rise in CLR (Q4: 46.4% vs. Q1: 43.1%; P = 0.033).

**Table 1:** Baseline characteristics of study participants stratified by CLR quartiles.

Characteristics	Overall (N = 5904)	Q1 (<0.588)	Q2 (≥0.588 to <1.058)	Q3 (≥1.058 to <1.815)	Q4 (≥1.815)	P-value
Age, years	51.4 ± 9.9	51.5 ± 9.9	50.8 ± 9.9	51.0 ± 9.8	52.3 ± 10.1	<0.001
Male sex	3204 (54.3%)	833 (56.4%)	846 (57.3%)	801 (54.3%)	724 (49.1%)	<0.001
Ethnicity						<0.001
Han	3795 (64.3%)	1087 (73.6%)	1004 (68.0%)	906 (61.4%)	798 (54.1%)	
Uyghur	1240 (21.0%)	190 (12.9%)	261 (17.7%)	351 (23.8%)	438 (29.7%)	
Other	869 (14.7%)	199 (13.5%)	211 (14.3%)	219 (14.8%)	240 (16.3%)	
Hypertension duration, months	80.0 ± 83.0	81.1 ± 85.6	77.6 ± 82.4	77.0 ± 79.0	84.3 ± 84.6	0.077
SBP, mmHg	144.8 ± 19.4	142.2 ± 19.0	144.1 ± 19.0	145.5 ± 19.5	147.4 ± 19.7	<0.001
DBP, mmHg	88.1 ± 14.2	86.6 ± 13.9	88.3 ± 14.1	88.4 ± 13.7	89.2 ± 14.8	<0.001
Uncontrolled Hypertension, n (%)	3856 (65.3%)	902 (61.1%)	938 (63.6%)	988 (66.9%)	1028 (69.6%)	<0.001
BMI, kg/m²	26.8 ± 3.7	25.8 ± 3.2	26.5 ± 3.4	27.2 ± 3.7	27.8 ± 4.2	<0.001
Diabetes mellitus	441 (7.5%)	100 (6.8%)	110 (7.5%)	108 (7.3%)	123 (8.3%)	0.445
Coronary artery disease	489 (8.3%)	120 (8.1%)	110 (7.5%)	113 (7.7%)	146 (9.9%)	0.067
Stroke	181 (3.1%)	42 (2.8%)	44 (3.0%)	48 (3.3%)	47 (3.2%)	0.915
Current smoking	1837 (31.1%)	476 (32.2%)	473 (32.0%)	457 (31.0%)	431 (29.2%)	0.259
Current drinking	1796 (30.4%)	447 (30.3%)	481 (32.6%)	473 (32.0%)	395 (26.8%)	0.002
Baseline eGFR, mL/min/1.73m²	105.74 ± 11.23	104.93 ± 11.04	106.60 ± 10.82	106.42 ± 10.75	105.01 ± 12.16	<0.001
Mild proteinuria (ACR 10-29.9 mg/g)	2580 (43.7%)	636 (43.1%)	652 (44.2%)	607 (41.1%)	685 (46.4%)	0.033
Uric acid, µmol/L	337.89 ± 90.02	325.83 ± 89.03	338.95 ± 87.56	341.65 ± 89.81	345.11 ± 92.54	<0.001
ALT, U/L	20.65 [15.00, 30.52]	19.00 [14.00, 28.00]	21.25 [15.00, 31.00]	20.85 [15.00, 31.00]	21.00 [15.00, 32.00]	<0.001
AST, U/L	18.00 [15.00, 22.00]	18.00 [15.00, 21.30]	18.00 [15.00, 22.00]	18.00 [15.00, 22.00]	18.00 [15.00, 23.00]	0.032
Fasting blood glucose, mmol/L	4.70 [4.30, 5.20]	4.60 [4.30, 5.10]	4.70 [4.30, 5.20]	4.80 [4.30, 5.20]	4.80 [4.40, 5.50]	<0.001
Total cholesterol, mmol/L	4.43 ± 0.99	4.30 ± 0.94	4.41 ± 1.01	4.47 ± 0.98	4.52 ± 1.02	<0.001
Triglycerides, mmol/L	1.50 [1.10, 2.10]	1.40 [1.00, 2.10]	1.50 [1.10, 2.10]	1.50 [1.10, 2.10]	1.50 [1.10, 2.10]	0.008
LDL-C, mmol/L	2.69 ± 0.85	2.56 ± 0.82	2.66 ± 0.85	2.73 ± 0.85	2.80 ± 0.86	<0.001
C-reactive protein, mg/L	2.92 ± 4.18	0.68 ± 0.39	1.63 ± 0.50	2.60 ± 0.87	6.76 ± 6.86	<0.001
Lymphocyte count, ×10^9^/L	1.92 ± 0.58	2.06 ± 0.63	2.00 ± 0.52	1.88 ± 0.56	1.74 ± 0.53	<0.001
CLR	1.69 ± 4.03	0.33 ± 0.15	0.82 ± 0.14	1.38 ± 0.21	4.22 ± 7.47	<0.001
ACEI/ARB	3230 (54.7%)	793 (53.7%)	790 (53.5%)	817 (55.4%)	830 (56.2%)	0.551
Beta-blockers	1285 (21.8%)	308 (20.9%)	298 (20.2%)	323 (21.9%)	356 (24.1%)	0.105
CCB	3536 (59.9%)	858 (58.1%)	885 (60.0%)	886 (60.0%)	907 (61.4%)	0.491
Spironolactone/MRA	323 (5.5%)	96 (6.5%)	76 (5.1%)	73 (4.9%)	78 (5.3%)	0.376
Statins	869 (14.7%)	229 (15.5%)	207 (14.0%)	213 (14.4%)	220 (14.9%)	0.835

Data are presented as mean ± standard deviation (SD) for continuous variables with normal distribution, median [interquartile range, IQR] for skewed continuous variables, or number (percentage) for categorical variables. P-values were derived from one-way analysis of variance (ANOVA) for normally distributed continuous variables, Kruskal-Wallis H test for skewed continuous variables, and Pearson’s chi-square test for categorical variables. ACEI, angiotensin-converting enzyme inhibitor; ARB, angiotensin receptor blocker; BMI, body mass index; CCB, calcium channel blocker; CLR, C-reactive protein-to-lymphocyte ratio; DBP, diastolic blood pressure; eGFR, estimated glomerular filtration rate; LDL-C, low-density lipoprotein cholesterol; MRA, mineralocorticoid receptor antagonist; SBP, systolic blood pressure.

### Cumulative incidence and risk trajectories

**Figure 2:**
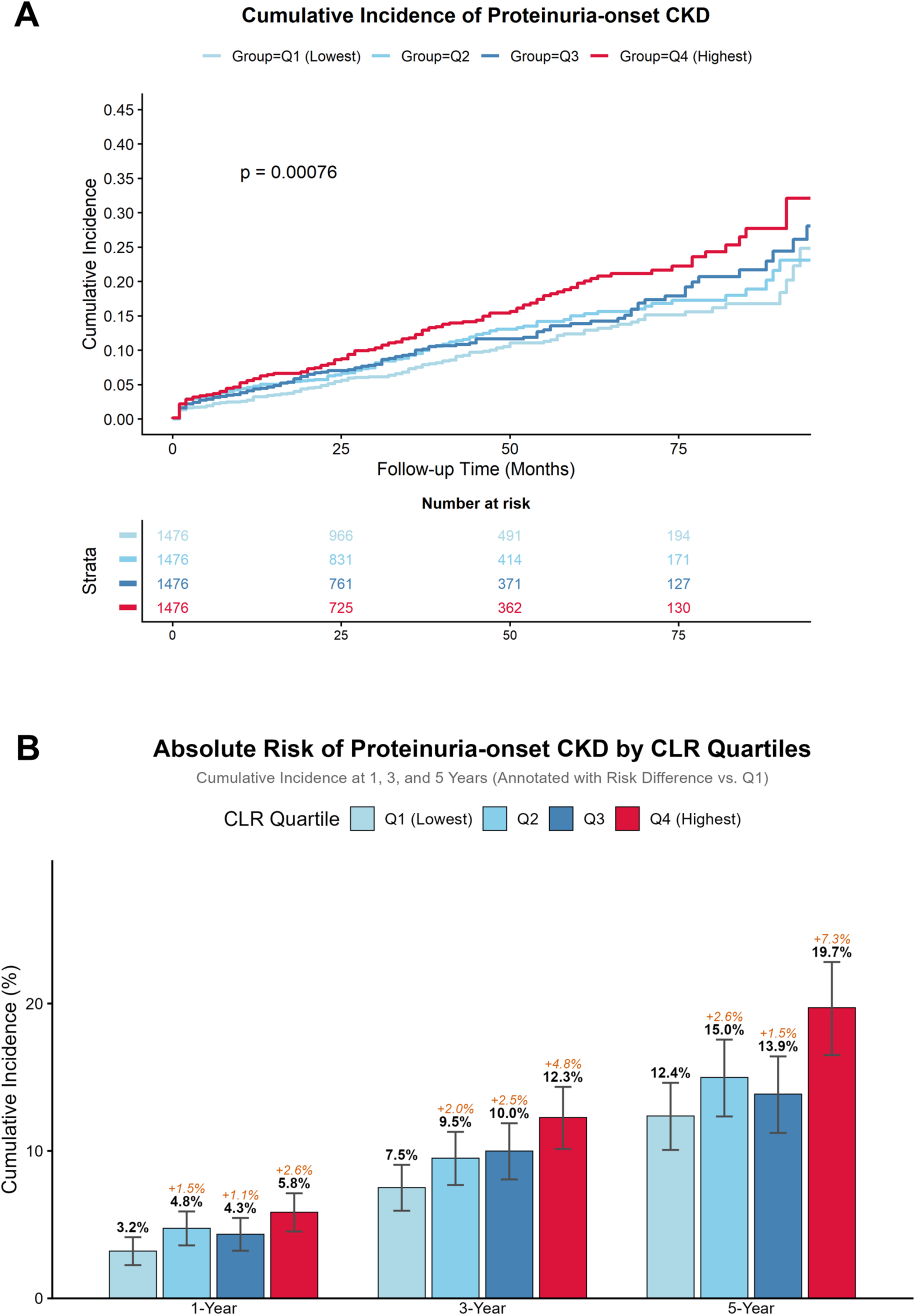
Cumulative incidence and absolute risk differences of proteinuric CKD stratified by CLR levels. **(A)** Kaplan-Meier cumulative incidence curves. **(B)** Absolute risk and risk differences.

Over a median follow-up of 34.1 months (totaling 16,678.2 person-years), we identified 728 cases of any incident CKD, encompassing 598 incident cases of proteinuric CKD and 89 cases of isolated eGFR decline. For the primary outcome of proteinuric CKD, the crude incidence rates exhibited a graded escalation across CLR quartiles, rising from 27.92 per 1,000 person-years in Q1 to 46.15 per 1,000 person-years in Q4 (**Supplementary Table 5**). Cumulative incidence analysis corroborated this trend, demonstrating a significantly elevated risk of proteinuric CKD in the highest CLR quartile over the follow-up period (Log-rank P < 0.001; **Figure 2A**).

This overall divergence between the highest and lowest quartiles (Q4 vs. Q1) corresponded to a time-dependent increase in absolute risk differences (ARD) of 2.6%, 4.8%, and 7.3% at 1, 3, and 5 years, respectively (**Figure 2B**), culminating in a 5-year cumulative incidence of 19.7% in Q4 versus 12.4% in Q1.

### Independent association with proteinuric CKD and dose-response

**Figure 3:**
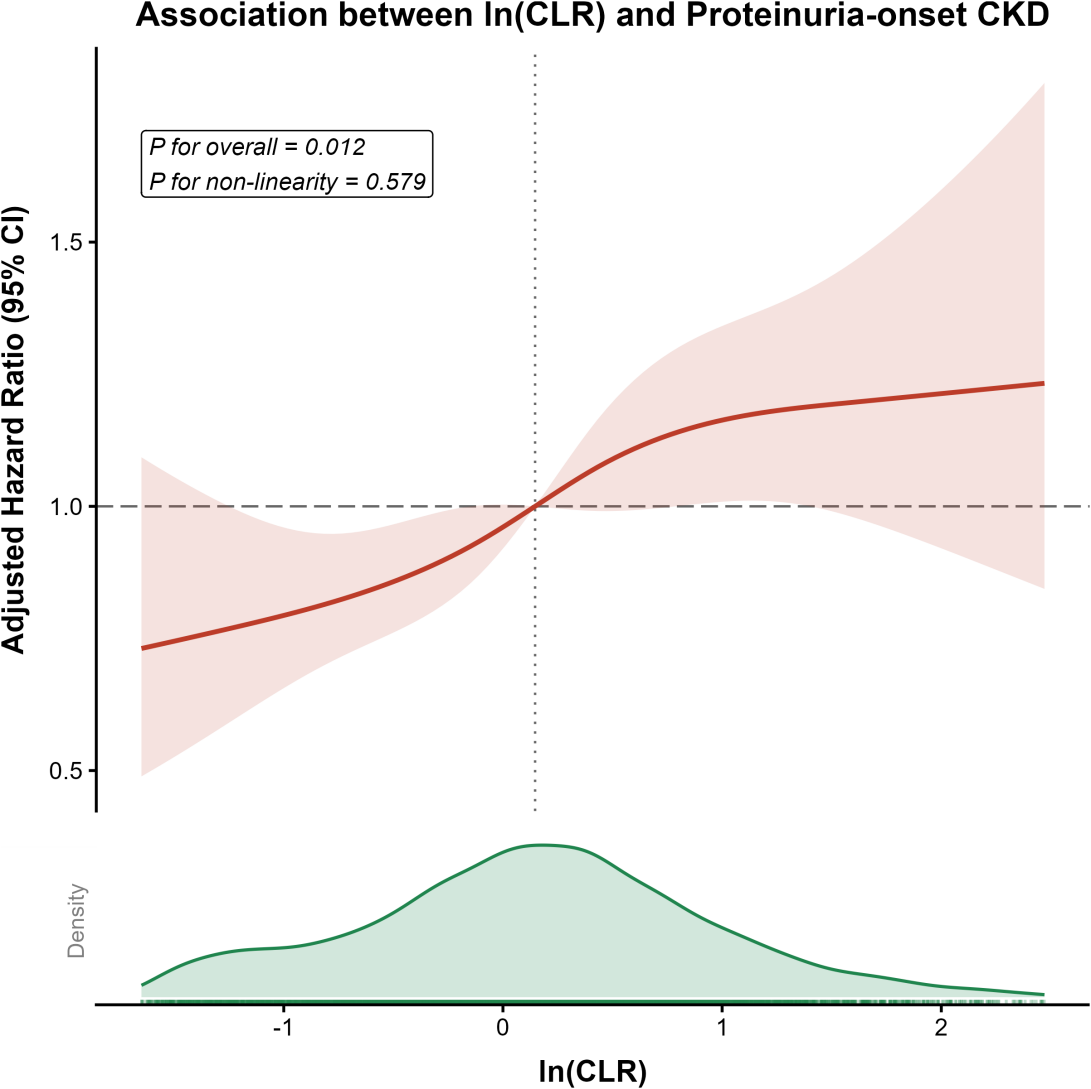
Dose-response relationship between baseline CLR and incident proteinuric CKD.

Multivariable Cox regression substantiated a robust, independent link between CLR and proteinuric CKD (**Table 2**). In the fully adjusted model (Model 3), each 1-SD increment in ln-transformed CLR conferred a 14% increase in the risk of proteinuric CKD (HR 1.14; 95% CI: 1.05–1.24; P = 0.001). Categorical analysis delineated a steep dose-response gradient (P for trend = 0.002): participants in Q4 faced a 46% higher risk relative to those in Q1 (HR 1.46; 95% CI: 1.15–1.85). Restricted cubic spline analysis (**Figure 3**) depicted this relationship as linear and monotonic (P for non-linearity = 0.579), with no discernible safety threshold.

**Table 2:** Independent association between baseline CLR and risk of incident proteinuric CKD.

Exposure	Model 1		Model 2		Model 3	
	HR (95% CI)	P value	HR (95% CI)	P value	HR (95% CI)	P value
Per 1-SD increase	1.19 (1.10-1.29)	<0.001	1.16 (1.07-1.26)	<0.001	1.14 (1.05-1.24)	0.001
Quartiles						
Q1 (Lowest)	Reference		Reference		Reference	
Q2	1.24 (0.98-1.56)	0.077	1.20 (0.95-1.52)	0.122	1.18 (0.93-1.49)	0.171
Q3	1.26 (0.99-1.60)	0.057	1.21 (0.95-1.53)	0.128	1.17 (0.92-1.49)	0.213
Q4 (Highest)	1.67 (1.33-2.10)	<0.001	1.54 (1.22-1.95)	<0.001	1.46 (1.15-1.85)	0.002
P for trend		<0.001		<0.001		0.002
High Risk Group						
Q1-Q3 (Reference)	Reference		Reference		Reference	
Q4 (Highest)	1.44 (1.20-1.72)	<0.001	1.36 (1.13-1.63)	0.001	1.31 (1.09-1.57)	0.004

Model 1: Adjusted for age, sex, and ethnicity. Model 2:Adjusted for covariates in Model 1 plus BMI, duration of hypertension, SBP, DBP, smoking status, drinking status, diabetes mellitus, coronary artery disease, and stroke. Model 3 (Fully Adjusted):Adjusted for covariates in Model 2 plus baseline eGFR, mild proteinuria (ACR 10–29.9 mg/g), uric acid, triglycerides, fasting blood glucose, LDL-C, and use of ACEI/ARBs, beta-blockers, calcium channel blockers, spironolactone/MRAs, and statins. CI, confidence interval; CLR, C-reactive protein-to-lymphocyte ratio; HR, hazard ratio; SD, standard deviation. *P for trend was calculated by entering the median value of each quartile as a continuous variable in the models.*

### Phenotype specificity

**Figure 4:**
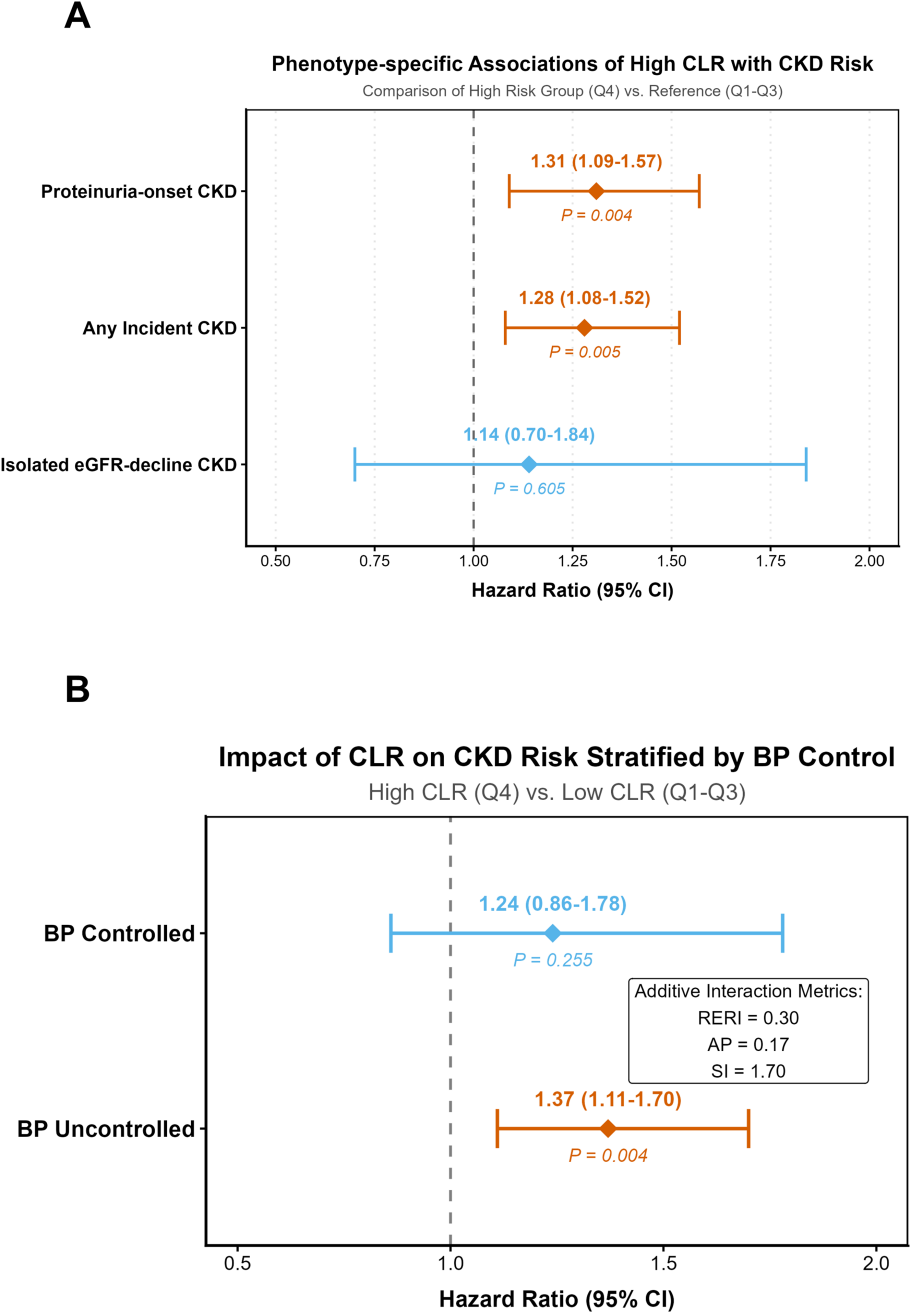
Phenotypic specificity of renal risk and synergistic interaction with baseline blood pressure. **(A)** Phenotype-specific risks. **(B)** Additive interaction with blood pressure control.

Incidence density analysis demonstrated that the composite adverse renal outcome was predominantly comprised of proteinuric events (35.86 per 1,000 person-years) rather than isolated filtration decline (5.09 per 1,000 person-years). Specifically, the incidence of proteinuric CKD progressively increased from 27.92 in Q1 to 46.15 per 1,000 person-years in Q4, whereas the incidence of isolated eGFR decline remained remarkably flat across all CLR quartiles (ranging from 4.75 to 5.99 per 1,000 person-years; **Supplementary Tables 5**-**S7**). Multivariable Cox models further confirmed that the prognostic value of elevated CLR was highly phenotype-specific (**Figure 4A**; **Table 3**). When comparing the highest risk group to the reference strata (Q4 vs. Q1–Q3), high CLR independently predicted an increased risk of both proteinuria-onset CKD (HR 1.31; 95% CI: 1.09–1.57) and any incident CKD (HR 1.28; 95% CI: 1.08–1.52). In contrast, CLR showed no significant association with isolated eGFR decline, regardless of whether it was modeled as a high-risk category (HR 1.14; 95% CI: 0.70–1.84) or as a continuous variable (per 1-SD increase: HR 1.05; 95% CI: 0.85–1.29).

**Table 3:** Phenotype-specific associations of CLR with renal outcomes using cause-specific Cox regression.

Exposure	Proteinuria-onset CKD		Isolated eGFR-decline CKD		Any Incident CKD	
	HR (95% CI)	P value	HR (95% CI)	P value	HR (95% CI)	P value
Per 1-SD increase	1.14 (1.05-1.24)	0.001	1.05 (0.85-1.29)	0.657	1.13 (1.05-1.22)	0.001
Quartiles						
Q1 (Reference)	1.00		1.00		1.00	
Q2	1.18 (0.93-1.49)	0.171	1.07 (0.59-1.93)	0.828	1.17 (0.94-1.46)	0.155
Q3	1.17 (0.92-1.49)	0.213	1.11 (0.61-2.05)	0.726	1.18 (0.94-1.48)	0.154
Q4 (Highest)	1.46 (1.15-1.85)	0.002	1.20 (0.67-2.18)	0.540	1.43 (1.15-1.79)	0.001
High Risk Group						
Q1-Q3 (Reference)	1.00		1.00		1.00	
Q4 (Highest)	1.31 (1.09-1.57)	0.004	1.14 (0.70-1.84)	0.605	1.28 (1.08-1.52)	0.005

Models: Proteinuria-onset CKDandAny Incident CKDmodels were adjusted for the fully adjusted Model 3 (see **Table 2** footer for details).Isolated eGFR-decline CKD model was adjusted for Model 2 only, adhering to the events per variable (EPV) principle due to the limited number of events (n=89). CI, confidence interval; CLR, C-reactive protein-to-lymphocyte ratio; eGFR, estimated glomerular filtration rate; HR, hazard ratio.

### Subgroup consistency and additive interaction

Subgroup analyses demonstrated broad consistency in the association between CLR and proteinuric CKD across all primary stratifications, including age, sex, BMI, diabetes status, and smoking status (all P-interaction > 0.05; **Supplementary Table 8**). Notably, this prognostic value remained robust regardless of baseline medication use. No significant effect modification was observed for the use of RAS inhibitors (P-interaction = 0.173) or statins (P-interaction = 0.550). suggesting that the predictive utility of CLR is not blunted by the potential pleiotropic anti-inflammatory effects of these agents (**Supplementary Table 8**). Beyond these uniform associations, a significant biological interaction emerged between systemic inflammation and BP control (**Figure 4B**; **Supplementary Table 9**). Analysis on the additive scale revealed a synergistic effect between high CLR (Q4 ≥ 1.815) and uncontrolled hypertension (SBP ≥ 140 mmHg and/or DBP ≥ 90 mmHg), with a RERI of 0.30, an AP of 0.17, and a SI of 1.70. Specifically, the independent prognostic value of high CLR was most pronounced in patients with uncontrolled BP (HR 1.37; 95% CI: 1.11–1.70; P = 0.004). In contrast, this association was attenuated to non-significance among those who achieved BP targets (HR 1.24; 95% CI: 0.86–1.78; P = 0.255), indicating that the pro-proteinuric impact of CLR is significantly modified by BP status.

### Predictive performance and risk reclassification

**Figure 5:**
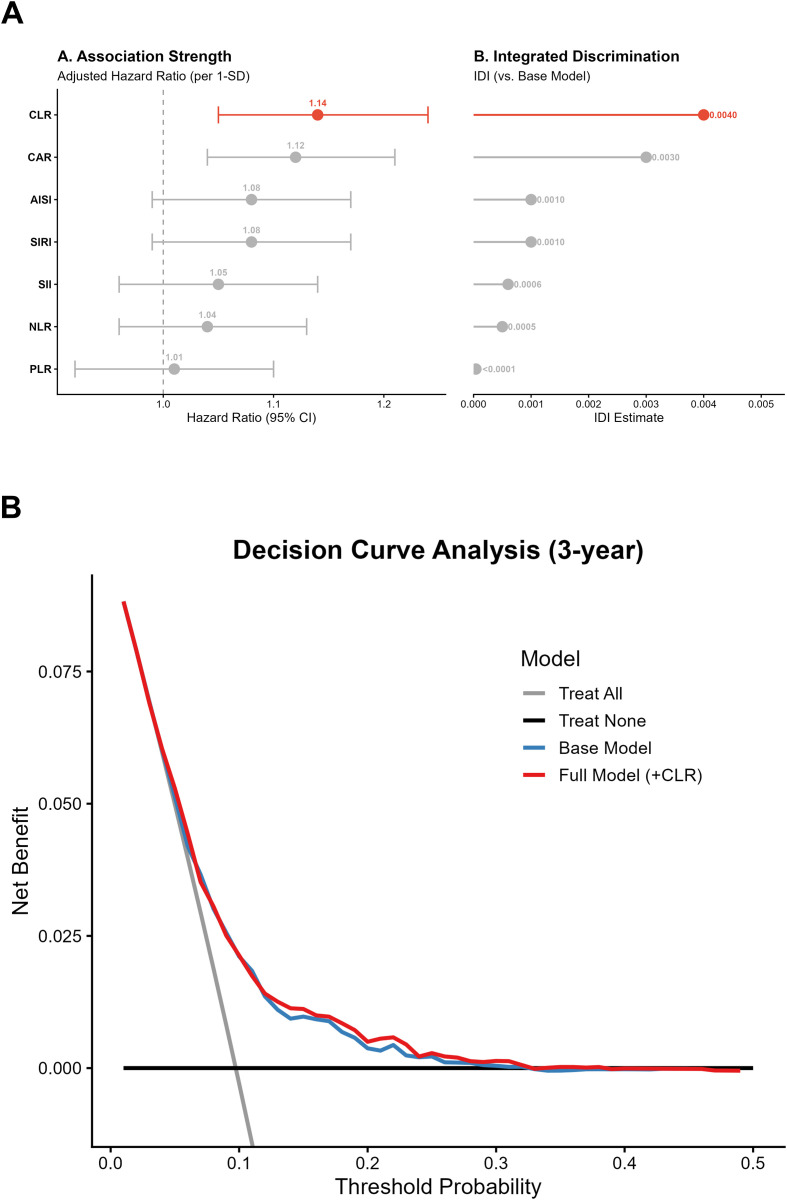
Comparative predictive efficacy and clinical decision utility of CLR. **(A)** Head-to-head comparison. **(B)** Decision curve analysis.

Among seven inflammatory indices evaluated, CLR demonstrated the strongest association with proteinuria-onset CKD and provided the greatest improvement in model discrimination (**Figure 5A**; **Supplementary Table 10**). The addition of CLR to the baseline clinical risk model yielded significant improvements in reclassification, with an NRI of 0.083 (P = 0.008) and an IDI of 0.004 (P = 0.047; **Table 4**). Calibration analysis showed high agreement between predicted and observed risks (O/E ratios: 0.98–1.08; **Supplementary Table 11**), and decision curve analysis confirmed higher net benefit at threshold probabilities between 15% and 40% (**Figure 5B**).

**Table 4:** Incremental value of adding CLR to traditional clinical risk factors for predicting proteinuric CKD.

Model	Harrell’s C-index	Δ C-index	1-year AUC	3-year AUC	5-year AUC	Continuous NRI (95% CI)	P value	IDI (95% CI)	P value
Traditional risk factors	0.653		0.659	0.654	0.678	Reference		Reference	
+ CLR (per 1-SD)	0.656	+0.002	0.667	0.660	0.680	0.083 (0.019-0.141)	0.008	0.004 (0.001-0.008)	0.047

Base model (traditional tisk factors):Includes age, sex, ethnicity, BMI, hypertension duration, SBP, DBP, smoking, drinking, diabetes, CVD history, baseline eGFR, mild proteinuria, uric acid, lipid profiles, and medication use (all covariates in Model 3 excluding CLR). Full model: Base Model + CLR (per 1-SD increase). Statistics: Harrell’s C-index: A measure of discrimination ability. NRI (Net Reclassification Improvement): Continuous NRI calculated at 3-year follow-up. IDI (Integrated Discrimination Improvement): Calculated at 3-year follow-up. 95% CIs and P-values for NRI and IDI were derived from 200 bootstrap resamples. AUC, area under the receiver operating characteristic curve; CI, confidence interval; CLR, C-reactive protein-to-lymphocyte ratio; SD, standard deviation.

Based on the Youden index, the optimal prognostic threshold for CLR was identified as 1.37. A baseline CLR ≥ 1.37 was associated with a 41% increased risk of incident proteinuric CKD (HR 1.41; 95% CI: 1.19–1.67; P < 0.001). Furthermore, individuals exceeding this threshold exhibited a significantly divergent cumulative incidence trajectory over the follow-up period (**Supplementary Figure 3**; **Supplementary Table 12**).

### Sensitivity and robustness analyses

The primary findings withstood a comprehensive battery of robustness checks (**Supplementary Table 13**-**S21**). The association between CLR and incident proteinuric CKD remained statistically significant and consistent in magnitude (adjusted HRs ~1.30–1.50) after: (1) excluding events within the first 3 or 6 months to minimize reverse causality; (2) excluding high-grade inflammation (CRP > 10 mg/L); (3) winsorizing CLR values at the 1st and 99th percentiles to limit the influence of extreme outliers; and (4) truncating the maximum follow-up to 3 years to address potential time-varying confounding and attrition. Furthermore, the results remained robust when analyses were restricted to non-smoking, non-drinking, and non-diabetic subgroups yielded unaltered conclusions. confirming the independence of this inflammatory risk axis. Finally, the calculated E-value for the primary estimate was 2.28, indicating that residual confounding could only explain away the observed effect if an unmeasured covariate was associated with both elevated CLR and incident proteinuric CKD by a risk ratio of at least 2.28, above and beyond the rigorously adjusted measured confounders.

## Discussion

To our knowledge, this study provides the first longitudinal evidence characterizing the phenotype-specific association between the CLR and renal injury in a hypertensive population. Utilizing a large-scale EHR-based cohort, we identified the CLR as a robust, independent predictor of incident CKD, with its prognostic power highly specific to proteinuric manifestations rather than isolated filtration decline. Furthermore, we identified a synergistic biological interaction on the additive scale between high CLR(Q4 ≥ 1.815) and uncontrolled hypertension, suggesting that inflammatory burden potentiates the microvascular damage precipitated by hemodynamic stress. Notably, the CLR demonstrated superior predictive performance compared to conventional inflammatory indices and provided significant incremental value for risk reclassification. These findings position the CLR as a reliable, cost-effective biomarker for quantifying renal risk and identifying high-risk individuals who may benefit from precision dual-management strategies.

The heterogeneity observed in prior studies regarding the inflammation-kidney axis partly stems from the undifferentiated use of composite outcomes (14, 15, 26, 27). This approach overlooks the distinct pathological trajectories of incident proteinuria and isolated eGFR decline during the early stages of hypertensive kidney damage (19, 20): the former is primarily driven by active microvascular barrier dysfunction and increased permeability (7, 21, 28), whereas the latter predominantly reflects cumulative nephron loss and ischemic sclerosis (29). A key methodological strength of our study is the application of cause-specific hazard models, which treat alternative or mixed renal phenotypes as censored events. This strategy effectively disentangles phenotypic heterogeneity and prevents non-inflammatory sclerotic pathways from diluting the prognostic signal, thereby confirming the core value of CLR in identifying early vascular barrier injury.

The phenotypic selectivity of CLR’s predictive value likely derives from a robust pathophysiological link between the systemic immune-inflammatory status it captures and the genesis of proteinuria. The immunological imbalance reflected by a high CLR (11, 30)—characterized by elevated CRP and concurrent lymphopenia—points to a milieu that disrupts both the charge and mechanical filtration barriers of the glomerulus (31, 32). This synergistic disruption is potentially driven by endothelial impairment associated with the acute-phase response and podocyte cytoskeletal rearrangement induced by systemic pro-inflammatory cytokines, providing a plausible pathophysiological basis for our phenotype-specific findings (22). While our real-world EHR-based design provides substantial epidemiological power, it inherently lacks specific molecular biomarkers to directly quantify the mediating effects on the endothelial and podocyte barriers. Future prospective biobank studies are warranted to elucidate these exact molecular mediating pathways.

The lack of association with isolated eGFR decline warrants nuanced interpretation. Beyond the possibility that the observation window was insufficient for anatomical sclerosis to fully manifest, this distinct observation may also be attributed to inflammation-associated glomerular hyperfiltration (33). During the early stages of inflammation, mediators such as prostaglandins can induce compensatory vasodilation of the afferent arterioles, maintaining or even elevating eGFR levels (34). This hemodynamic compensation may mask the early insidious injury to the nephrons. However, this observation reinforces the prognostic value of CLR in monitoring inflammation-mediated early renal injury.

Furthermore, the dose-response analysis revealed a linear, dose-dependent trajectory with no discernible safety threshold, implying that even “high-normal” micro-inflammation exerts continuous deleterious stress on an endothelium already compromised by hemodynamic load (35, 36). Crucially, this association remained robust in the non-diabetic subgroup.This robustness is of particular clinical relevance, as it confirms that CLR captureshypertension-specific inflammation, rather than reflecting pathways driven solely by hyperglycemic “metabolic memory” (37, 38).

To quantify this pervasive background risk, our comparative analysis identified CLR as the most robust prognostic marker among the evaluated inflammatory indices. Furthermore, the significant improvements in reclassification metrics (NRI and IDI) indicate that incorporating CLR into established risk models provides meaningful incremental prognostic value. To operationalize this enhanced risk stratification, a baseline CLR cutoff of ≥ 1.37 could serve as a practical early-warning threshold for the general hypertensive population. Utilizing this readily available index aids clinicians in proactively identifying individuals who require vigilant surveillance for incident proteinuria alongside routine BP management, thereby facilitating the early detection of insidious filtration barrier injury. Crucially, from the broader perspective of CKD prevention, the core clinical value of CLR lies in quantifying the “non-hemodynamic residual risk” that traditional management paradigms inadequately address. Indeed, the significant additive interaction between high CLR and uncontrolled blood pressure strongly suggests a biological interaction, reflecting a synergistic pathogenic mechanism where hypertension-driven mechanical stress and a systemic pro-inflammatory microenvironment jointly compromise the renal microvasculature (39, 40). Our stratified data clearly corroborate this: when BP is optimally controlled, the pro-proteinuric effect of high CLR is markedly attenuated. However, when a patient presents with a baseline CLR in the highest quartile (≥ 1.815) alongside uncontrolled BP, their risk for future proteinuria-onset kidney injury is synergistically amplified due to the concurrent insults of mechanical stress and inflammatory toxicity. For these highest-risk patients with severe baseline inflammatory burdens, sole reliance on intensive BP-lowering may yield diminishing marginal benefits over time. Consequently, future clinical management for such individuals might incorporate proactive anti-inflammatory or endothelium-protective therapies.

Our study benefits from a large sample size, a longitudinal design, and a rigorous phenotype-specific framework. However, several methodological constraints merit transparent discussion. First, as an observational study, despite extensive multivariable adjustment and E-value analysis, residual confounding (e.g., dietary salt intake, genetic susceptibility) cannot be entirely eliminated, nor can definitive causality be established. Second, relying on real-world EHR data inherently introduces selection bias, leaving the analysis susceptible to surveillance bias and informative censoring. However, by strictly applying right-censoring at the date of the last valid clinical encounter—rather than assuming event-free survival for lost patients—and demonstrating robust consistency in our sensitivity analyses (e.g., truncating follow-up at 3 years), we minimized the potential for differential attrition bias. Third, the single-center retrospective design inherently limits the external generalizability of our overall findings. While the multi-ethnic demographic of the Xinjiang region provides a degree of diversity often lacking in homogenous cohorts—and our supplementary subgroup analysis (detailed in the Notes of **Supplementary Table 12**) demonstrated consistent prognostic performance across these distinct ethnic backgrounds without significant effect modification (P for interaction = 0.514)—this regional robustness does not equate to universal applicability. The magnitude of the observed associations and the optimal CLR risk thresholds may vary across different geographical regions and demographic characteristics. Consequently, future external validation in multi-center, international cohorts with diverse healthcare systems remains necessary to confirm the universal applicability of our findings and specific risk thresholds. Fourth, the lack of repeated CLR measurements prevents us from evaluating time-varying effects. Fifth, inherent limitations in EHR-based outcome ascertainment must be acknowledged. Primarily, the irregular nature of real-world clinical encounters precluded the universal verification of the KDIGO chronicity criterion (i.e., sustained abnormalities over 3 months). Furthermore, when quantitative proteinuria assessments were unavailable, we relied on automated semi-quantitative readings. Because these are predominantly derived from morning or random spot urine samples, they are susceptible to false-positive misclassifications. To strictly counterbalance this measurement vulnerability, we mandated a conservative diagnostic threshold of ≥ 2+ (rather than the conventional ≥ 1+). By explicitly prioritizing diagnostic specificity, this stringent definition minimizes the inadvertent capture of transient proteinuria driven by benign physiological or hemodynamic fluctuations, ensuring the identified events reflect genuine structural injury. Sixth, our “phenotypes” were defined by clinical criteria rather than histology, precluding direct confirmation of specific lesions. Finally, the unavailability of all-cause mortality data precluded a competing risk analysis. Nevertheless, within our cause-specific hazard framework, attrition due to unrecorded mortality is implicitly handled as right-censoring at the last valid clinical encounter; while this precludes estimating absolute risk in the presence of competing events, it remains a standard and valid approach for deriving unbiased etiological hazard ratios (41).

In hypertensive patients, elevated CLR is an independent, phenotype-specific predictor of incident proteinuric CKD, but not isolated eGFR decline. Beyond serving as an accessible and cost-effective biomarker for renal risk stratification, CLR identifies a profound synergistic interaction with uncontrolled blood pressure that severely amplifies the risk of microvascular injury. These findings highlight a vulnerable sub-population that requires vigilant proteinuria surveillance and may benefit from prompt ‘precision dual-management’ targeting both hemodynamic stress and residual inflammatory burden. Finally, rigorous external validation in multi-center prospective cohorts is imperative to confirm the generalizability of these findings, and randomized controlled trials are warranted to determine whether CLR-guided interventions can effectively mitigate renal disease progression.

## Data Availability

The raw data supporting the conclusions of this article will be made available by the authors, without undue reservation.
