# Supplementary material for: Phenotype-specific association of C-reactive protein-to-lymphocyte ratio with incident proteinuric CKD versus isolated eGFR decline: a real-world retrospective cohort study
Source: Front Endocrinol (Lausanne). 2026 Apr 1;17:1769059. doi: 10.3389/fendo.2026.1769059 (PMC13079130; doi:10.3389/fendo.2026.1769059)

**Supplementary** **Methods**

**S1. Detailed Exclusion Criteria**
To ensure cohort homogeneity and minimize residual confounding, we implemented a rigorous, multi-step exclusion protocol based on the following specific criteria:

**1. Baseline Renal Pathology and Anatomical Abnormalities**
Beyond the primary exclusion of baseline eGFR < 60 mL/min/1.73 m² or evidence of pathological proteinuria (ACR ≥ 30 mg/g, 24-h urine protein ≥ 150 mg, or automated semi-quantitative protein ≥ 1+), we further excluded patients with a documented history of:

- **Primary/Secondary Glomerular Diseases:** Including glomerulonephritis, nephrotic syndrome, IgA nephropathy.
- **Congenital or Structural Anomalies:** Including polycystic kidney disease, solitary kidney, or history of nephrectomy.
- **Renovascular and Obstructive Lesions:** Including renal artery stenosis, obstructive nephropathy, hydronephrosis, and significant renal atrophy.

**2. Acute Inflammation and Infectious Diseases**
To prevent acute-phase reactions from masking the true baseline CLR-risk association, patients presenting with the following acute infections upon admission were excluded:

- **Respiratory Infections:** Acute upper respiratory tract infection, pneumonia, and bronchitis.
- **Urinary Tract Infections:** Acute pyelonephritis, cystitis, and symptomatic bacteriuria.
- **Abdominal and Surgical Emergencies:** Acute appendicitis, cholecystitis, peritonitis, and acute gastrointestinal infections.
- **Other Acute Infections:** Including sepsis and active skin/soft tissue infections.

**3. Malignancies and Autoimmune Diseases**
We excluded conditions that could alter the inflammation-kidney axis via tumor burden or immunosuppressive therapy:

- **Active Malignancies:** Solid tumors and hematological malignancies (e.g., leukemia, lymphoma, multiple myeloma).
- **Autoimmune and Connective Tissue Diseases:** Systemic lupus erythematosus (SLE), rheumatoid arthritis, systemic vasculitis, Sjögren's syndrome, ankylosing spondylitis, and Behçet's disease.
- **Immunocompromised Status:** Chronic use of systemic corticosteroids or immunosuppressants (e.g., cyclophosphamide, cyclosporine, tacrolimus).

**4. Severe Systemic Diseases and Extreme Physiological States**

- **Cachexia and Heart Failure:** Clinically diagnosed cachexia or severe heart failure (NYHA Class III-IV).
- **Acute Cardiovascular Events:** Acute myocardial infarction, unstable angina, or stroke occurring within 3 months prior to admission.
- **Severe Hepatic Impairment:** Defined as baseline ALT or AST levels > 3 times the upper limit of normal (ULN).

**S2. Formulas for Inflammatory Indices**
The primary exposure, C-reactive Protein-to-Lymphocyte Ratio (CLR), and six comparative inflammatory indices were calculated based on fasting venous blood samples. The formulas are as follows:

1. **C-reactive Protein-to-Lymphocyte Ratio (CLR):** CLR = CRP (mg/L) / Lymphocyte count (10⁹/L)
2. **Neutrophil-to-Lymphocyte Ratio (NLR):** NLR = Neutrophil count / Lymphocyte count
3. **Platelet-to-Lymphocyte Ratio (PLR):** PLR = Platelet count / Lymphocyte count
4. **Systemic Immune-Inflammation Index (SII):** SII = (Platelet count × Neutrophil count) / Lymphocyte count
5. **Systemic Inflammation Response Index (SIRI):** SIRI = (Neutrophil count × Monocyte count) / Lymphocyte count
6. **Aggregate Index of Systemic Inflammation (AISI):** AISI = (Neutrophil count × Platelet count × Monocyte count) / Lymphocyte count
7. **C-reactive Protein-to-Albumin Ratio (CAR):** CAR = CRP (mg/L) / Albumin (g/L)

**S3. Detailed Statistical Methodology**

**(1) Missing Data Handling and Distributional Transformation**
The proportion of missing data for baseline covariates was < 12%. Under the assumption of Missing At Random (MAR), we employed Multiple Imputation by Chained Equations (MICE) using the *mice* package in R. We generated 10 imputed datasets with 20 iterations using Predictive Mean Matching (PMM). Sensitivity analyses revealed that the covariate distribution in the imputed datasets was highly consistent with the original data (Table S1).

Although multiple imputed datasets were generated, all primary multivariable regression analyses and complex predictive models (e.g., restricted cubic splines, decision curve analysis, NRI, and IDI) were performed using the first imputed dataset. This approach was chosen due to computational limitations with advanced predictive analysis R packages, which do not fully support pooled multiple imputation datasets (as per Rubin's rules). This method is justified given the relatively low overall missing data rate and the negligible differences observed between pre- and post-imputation data distributions (Table S1).
Given the highly right-skewed distribution of raw CLR (skewness = 32.17), a natural logarithmic transformation (ln-CLR) followed by Z-score standardization was applied. Post-transformation, the skewness of ln[CLR] was reduced to 0.33, demonstrating a satisfactory approximation to a normal distribution (Figure S1 & Table S2).

**(2) Verification of Model Assumptions**

- **Multicollinearity:** Variance Inflation Factors (VIF) were calculated for all covariates. In the fully adjusted Model 3, all VIF values were < 5, indicating no severe multicollinearity (**Table S3**).
- **Proportional Hazards (PH) Assumption:** The PH assumption was tested using scaled Schoenfeld residuals. Although the global test showed nominal statistical significance (P=0.040)—a phenomenon often driven by large sample sizes and high statistical power—the key exposure variable, CLR, fully satisfied the PH assumption (P=0.331). Visual inspection of the scaled Schoenfeld residual plots confirmed no obvious non-linear patterns over time (**Figure S2**).

**(3) Assessment of Biological Interaction (Additive Scale)**
To evaluate the synergistic effect between high CLR (Q4) and poor blood pressure control, we calculated indices of interaction on the additive scale using the method proposed by Andersson et al^[1]^., along with their 95% confidence intervals:

- **Relative Excess Risk due to Interaction (RERI):** RERI = *HR_11_* – *HR_10_* – *HR_01_* + 1
- **Attributable Proportion (AP):** AP = *RERI / HR_11_*
- **Synergy Index (SI):** *SI = (HR_11_ – 1) / ((HR_10_ – 1) + (HR_01_ – 1))*

*Interpretation: HR_11_* represents the hazard ratio for the double-exposed group (High CLR + Uncontrolled BP). A result of *RERI* > 0, *AP* > 0, or *SI* > 1 indicates a positive additive interaction (synergy).

**(4) Algorithmic Specifications for Predictive Metrics**

To rigorously quantify the incremental prognostic value of CLR beyond traditional risk factors (Model 3), we utilized the following metrics: Harrell’s C-index and Time-dependent AUC: Used to evaluate overall model discrimination and dynamic predictive accuracy over the follow-up period. Continuous Net Reclassification Improvement (NRI) and Integrated Discrimination Improvement (IDI): Used to quantify the correct reclassification of patients' risk probabilities upon the addition of CLR. To ensure internal validity and reduce overfitting bias, the 95% confidence intervals (CIs) for NRI and IDI were derived from 200 bootstrap resamples. Decision Curve Analysis (DCA): Employed to estimate the clinical net benefit of the CLR-integrated model across varying threshold probabilities, with the optimal risk-stratification cutoff identified mathematically via the Youden index.

**S4. Sensitivity Analysis for Unmeasured Confounding (E-value)**
To quantify the potential impact of unmeasured confounding on our primary findings, we calculated the E-value using the methodology described by VanderWeele and Ding^[2]^. The E-value represents the minimum strength of association that an unmeasured confounder would need to have with both the exposure (CLR) and the outcome (proteinuric CKD) to explain away the observed exposure-outcome association, conditional on the measured covariates.

**Reference：**

1. Knol MJ, VanderWeele TJ. Recommendations for presenting analyses of effect modification and interaction. Int J Epidemiol. 2012 Apr;41(2):514-20. doi: 10.1093/ije/dyr218. Epub 2012 Jan 9.
2. VanderWeele TJ, Ding P. Sensitivity Analysis in Observational Research: Introducing the E-Value. Ann Intern Med. 2017 Aug 15;167(4):268-274. doi: 10.7326/M16-2607. Epub 2017 Jul 11.

**Supplemental Table S1. Comparison of Baseline Characteristics Between the Original Dataset and the Imputed Dataset**

| Variable | Missing Count (N) | Missing Rate (%) | Observed Data (Before Imputation) | Imputed Data (After Imputation) |
| --- | --- | --- | --- | --- |
| Age, years | 0 | 0.0% | 51.39 ± 9.94 | 51.39 ± 9.94 |
| Gender (Male) n (%) | 0 | 0.0% | 3204 (54.3%) | 3204 (54.3%) |
| Ethnicity (Han ethnicity) n (%) | 0 | 0.0% | 3795 (64.3%) | 3795 (64.3%) |
| BMI, kg/m² | 195 | 3.3% | 26.83 ± 3.69 | 26.81 ± 3.70 |
| Hypertension duration months | 378 | 6.4% | 81.15 ± 83.49 | 80.02 ± 82.98 |
| SBP, mmHg | 5 | 0.1% | 144.80 ± 19.36 | 144.82 ± 19.37 |
| DBP, mmHg | 6 | 0.1% | 88.12 ± 14.15 | 88.13 ± 14.16 |
| Current smoking, n (%) | 0 | 0.0% | 1837 (31.1%) | 1837 (31.1%) |
| Current drinking, n (%) | 0 | 0.0% | 1796 (30.4%) | 1796 (30.4%) |
| Diabetes, n (%) | 0 | 0.0% | 441 (7.5%) | 441 (7.5%) |
| Coronary artery disease, (%) | 0 | 0.0% | 489 (8.3%) | 489 (8.3%) |
| Stroke, n (%) | 0 | 0.0% | 181 (3.1%) | 181 (3.1%) |
| Baseline eGFR, mL/min/1.73m² | 0 | 0.0% | 105.74 ± 11.23 | 105.74 ± 11.23 |
| Urine ACR, mg/g | 653 | 11.1% | 10.41 ± 5.40 | 10.45 ± 5.40 |
| Uric acid, µmol/L | 6 | 0.1% | 337.95 ± 90.00 | 337.89 ± 90.02 |
| Triglycerides, mmol/L | 38 | 0.6% | 1.77 ± 1.26 | 1.76 ± 1.25 |
| Fasting blood glucose, mmol/L | 677 | 11.5% | 4.98 ± 1.31 | 5.00 ± 1.32 |
| LDL-C, mmol/L | 38 | 0.6% | 2.69 ± 0.85 | 2.69 ± 0.85 |
| ACEI/ARB use, n (%) | 0 | 0.0% | 3230 (54.7%) | 3230 (54.7%) |
| Beta-blocker use, n (%) | 21 | 0.4% | 1283 (21.8%) | 1285 (21.8%) |
| CCB use, n (%) | 1 | 0.0% | 3535 (59.9%) | 3536 (59.9%) |
| MRA/Spironolactone use, n (%) | 0 | 0.0% | 323 (5.5%) | 323 (5.5%) |
| Statin use, n (%) | 0 | 0.0% | 869 (14.7%) | 869 (14.7%) |
| C-reactive protein, mg/L | 0 | 0.0% | 2.92 ± 4.18 | 2.92 ± 4.18 |
| Lymphocyte count, ×10⁹/L | 0 | 0.0% | 1.92 ± 0.58 | 1.92 ± 0.58 |
| Neutrophil count, ×10⁹/L | 0 | 0.0% | 3.62 ± 1.24 | 3.62 ± 1.24 |
| Monocyte count, ×10⁹/L | 0 | 0.0% | 0.43 ± 0.15 | 0.43 ± 0.15 |
| Platelet count, ×10⁹/L | 3 | 0.1% | 240.27 ± 60.16 | 240.29 ± 60.18 |
| Albumin, g/L | 623 | 10.6% | 41.20 ± 3.24 | 41.21 ± 3.24 |

**Abbreviations:** BMI, body mass index; SBP, systolic blood pressure; DBP, diastolic blood pressure; eGFR, estimated glomerular filtration rate; SD, standard deviation.

**Supplemental Table S2. Distributional Characteristics of C-Reactive Protein-to-Lymphocyte Ratio (CLR) Before and After Transformation**

| Variable | Mean ± SD | Median [IQR] | Range (Min-Max) | Skewness | Kurtosis | P for normality |
| --- | --- | --- | --- | --- | --- | --- |
| Raw CLR | 1.69 ± 4.03 | 1.06 [0.59, 1.81] | 0.01 - 223.68 | 32.17 | 1609.53 | <0.001 |
| ln(CLR) | 0.15 ± 0.85 | 0.15 [-0.37, 0.65] | -2.18 - 5.41 | 0.33 | 0.93 | <0.001 |
| **Abbreviations:** CLR, C-reactive protein-to-lymphocyte ratio; SD, standard deviation. | | | | | | |

**Supplemental Table S3. Collinearity Diagnostics for Covariates Included in the Fully Adjusted Multivariable Model**

| Variable | Step 1: VIF (All Variables) | Step 2: VIF (Final Model) |
| --- | --- | --- |
| Total cholesterol | 7.78 | - |
| LDL-C | 6.87 | 1.12 |
| DBP | 2.56 | 2.54 |
| Age | 2.44 | 2.39 |
| Gender | 2.29 | 2.23 |
| Triglycerides | 2.27 | 1.17 |
| SBP | 2.22 | 2.22 |
| Current smoking | 1.97 | 1.95 |
| Current drinking | 1.89 | 1.88 |
| Baseline eGFR | 1.88 | 1.87 |
| Uric acid | 1.73 | 1.71 |
| Hypertension duration | 1.37 | 1.37 |
| BMI | 1.31 | 1.27 |
| FBG | 1.29 | 1.28 |
| Ethnicity | 1.26 | 1.21 |
| Diabetes | 1.24 | 1.23 |
| Statin use | 1.21 | 1.21 |
| ACEI/ARB use | 1.16 | 1.16 |
| CCB use, | 1.16 | 1.15 |
| Lymphocyte count | 1.12 | - |
| Beta-blocker use | 1.11 | 1.11 |
| Coronary artery disease | 1.10 | 1.1 |
| Mild proteinuria | 1.10 | 1.1 |
| C-reactive protein | 1.06 | - |
| MRA/Spironolactone use | 1.04 | 1.04 |
| Stroke | 1.03 | 1.03 |
| **Notes:** **Threshold:** A VIF value < 5 indicates no severe multicollinearity. In Step 1, variables with high VIF (e.g., Total cholesterol, LDL-C) were identified. In the final model (Step 2), redundancy was resolved (e.g., by retaining LDL-C and removing Total Cholesterol/CRP/Lymphocytes which are components of other variables), ensuring all final VIF values were < 5. **Abbreviations:** ACEI, angiotensin-converting enzyme inhibitor; ARB, angiotensin receptor blocker; BMI, body mass index; CCB, calcium channel blocker; DBP, diastolic blood pressure; eGFR, estimated glomerular filtration rate; FBG, fasting blood glucose; LDL-C, low-density lipoprotein cholesterol; MRA, mineralocorticoid receptor antagonist; SBP, systolic blood pressure. | | |

**Supplemental Table S4. Test of Proportional Hazards Assumption Based on Schoenfeld Residuals**

| Variable | Chi-Square | P value |
| --- | --- | --- |
| Ln-transformed CLR (per 1-SD) | 0.95 | 0.331 |
| Age | 3.74 | 0.053 |
| Sex | 5.05 | 0.025 |
| Ethnicity | 1.70 | 0.427 |
| Body mass index | 0.02 | 0.885 |
| Hypertension duration | 0.03 | 0.857 |
| Systolic blood pressure | 0.02 | 0.897 |
| Diastolic blood pressure | 1.81 | 0.179 |
| Current smoking | 3.12 | 0.077 |
| Current drinking | 0.54 | 0.463 |
| Diabetes mellitus | 0.44 | 0.506 |
| Coronary artery disease | 5.31 | 0.021 |
| Stroke | 3.05 | 0.081 |
| Baseline eGFR | 1.50 | 0.220 |
| Mild proteinuria | 0.40 | 0.525 |
| Uric acid | 3.18 | 0.075 |
| Triglycerides | 0.61 | 0.433 |
| Fasting blood glucose | 1.40 | 0.237 |
| LDL-C | 4.06 | 0.044 |
| ACEI/ARB | 0.08 | 0.772 |
| Beta-blockers | 1.33 | 0.248 |
| Calcium channel blockers | 1.26 | 0.261 |
| Spironolactone/MRA | 2.61 | 0.106 |
| Statins | 3.99 | 0.046 |
| Global Test | 38.61 | 0.040 |

**Notes:**
The proportional hazards (PH) assumption was statistically tested using the Grambsch-Therneau test based on scaled Schoenfeld residuals.
**Interpretation:** A P-value > 0.05 indicates that the PH assumption is not violated.
**Abbreviations:** ACEI, angiotensin-converting enzyme inhibitor; ARB, angiotensin receptor blocker; CLR, C-reactive protein-to-lymphocyte ratio; LDL-C, low-density lipoprotein cholesterol; MRA, mineralocorticoid receptor antagonist; SD, standard deviation.

**Supplemental Table S5. Incidence Rates and Cumulative Incidence of Proteinuria-Onset CKD According to CLR Quartiles**

| CLR Quartile | No. of Participants | No. of Events | Person-years | Incidence Rate (per 1000 person-years) (95% CI) | 3-year Cumulative Incidence (%) |
| --- | --- | --- | --- | --- | --- |
| Overall | 5904 | 598 | 16678.2 | 35.86 (33.04-38.85) | 9.7% |
| Q1 | 1476 | 133 | 4763.2 | 27.92 (23.38-33.09) | 7.5% |
| Q2 | 1476 | 149 | 4199.9 | 35.48 (30.01-41.65) | 9.5% |
| Q3 | 1476 | 142 | 3944.4 | 36.00 (30.32-42.43) | 10.0% |
| Q4 | 1476 | 174 | 3770.7 | 46.15 (39.54-53.53) | 12.3% |
| **Notes:** Incidence Rate: Calculated as the number of events divided by the total person-years of follow-up, expressed per 1,000 person-years. 3-Year Cumulative Incidence: Derived from Kaplan-Meier estimates. P-value: Derived from the Log-rank test comparing survival curves across quartiles. **Abbreviations:** CI, confidence interval; CKD, chronic kidney disease; CLR, C-reactive protein-to-lymphocyte ratio. | | | | | |

***Supplemental Table S6. Incidence Rates and Cumulative Incidence of Isolated eGFR-Decline CKD According to CLR Quartiles***

| CLR Quartile | No. of Participants | No. of Events | Person-years | Incidence Rate (per 1000 person-years) (95% CI) | 3-year Cumulative Incidence (%) |
| --- | --- | --- | --- | --- | --- |
| Overall | 5904 | 89 | 17495.9 | 5.09 (4.09-6.26) | 1.5% |
| Q1 | 1476 | 24 | 4955.8 | 4.84 (3.10-7.21) | 1.4% |
| Q2 | 1476 | 21 | 4417.8 | 4.75 (2.94-7.27) | 1.5% |
| Q3 | 1476 | 20 | 4115.2 | 4.86 (2.97-7.51) | 1.4% |
| Q4 | 1476 | 24 | 4007.2 | 5.99 (3.84-8.91) | 1.8% |

**Abbreviations:** CI, confidence interval; CKD, chronic kidney disease; CLR, C-reactive protein-to-lymphocyte ratio; eGFR, estimated glomerular filtration rate.

**Supplemental Table S7. Incidence Rates and Cumulative Incidence of Any Incident CKD According to CLR Quartiles**

| CLR Quartile | No. of Participants | No. of Events | Person-years | Incidence Rate (per 1000 person-years) (95% CI) | 3-year Cumulative Incidence (%) |
| --- | --- | --- | --- | --- | --- |
| Overall | 5904 | 728 | 16440 | 44.28 (41.12-47.62) | 12.2% |
| Q1 | 1476 | 177 | 4689.1 | 37.75 (32.39-43.74) | 10.3% |
| Q2 | 1476 | 171 | 4156.5 | 41.14 (35.21-47.79) | 11.5% |
| Q3 | 1476 | 174 | 3896.1 | 44.66 (38.27-51.81) | 12.2% |
| Q4 | 1476 | 206 | 3698.3 | 55.70 (48.35-63.85) | 15.2% |
| P value for Log-rank test: 0.003 | | | | | |

**Abbreviations:** CI, confidence interval; CKD, chronic kidney disease; CLR, C-reactive protein-to-lymphocyte ratio.

**Supplemental Table S8. Subgroup Analysis of the Association Between High CLR and Proteinuria-Onset CKD**

| Subgroup |  | No. of Patients | Events n (%) | Adjusted HR (95% CI) (Q4 vs Q1-Q3) | P value | P for interaction |
| --- | --- | --- | --- | --- | --- | --- |
| Gender | Female | 2700 | 226 (8.4%) | 1.27 (0.95-1.70) | 0.113 | 0.638 |
|  | Male | 3204 | 372 (11.6%) | 1.29 (1.01-1.63) | 0.040 |  |
| Age, years | <60 years | 4638 | 495 (10.7%) | 1.25 (1.02-1.53) | 0.035 | 0.572 |
|  | ≥60 years | 1266 | 103 (8.1%) | 1.51 (0.98-2.33) | 0.061 |  |
| BMI, kg/m² | BMI <25 kg/m² | 1905 | 156 (8.2%) | 1.10 (0.74-1.65) | 0.633 | 0.334 |
|  | BMI ≥25 kg/m² | 3999 | 442 (11.1%) | 1.37 (1.12-1.69) | 0.003 |  |
| Diabetes Mellitus | No | 5463 | 547 (10.0%) | 1.28 (1.06-1.56) | 0.011 | 0.337 |
|  | Yes | 441 | 51 (11.6%) | 1.90 (0.99-3.64) | 0.052 |  |
| Baseline eGFR | eGFR ≥90 | 5399 | 544 (10.1%) | 1.29 (1.06-1.57) | 0.009 | 0.953 |
|  | eGFR <90 | 505 | 54 (10.7%) | 1.46 (0.77-2.77) | 0.245 |  |
| Smoking status | No | 4067 | 383 (9.4%) | 1.43 (1.14-1.79) | 0.002 | 0.218 |
|  | Yes | 1837 | 215 (11.7%) | 1.14 (0.83-1.57) | 0.432 |  |
| ACEI/ARBs use | No | 2674 | 216 (8.1%) | 1.05 (0.76-1.46) | 0.760 | 0.173 |
|  | Yes | 3230 | 382 (11.8%) | 1.44 (1.15-1.80) | 0.001 |  |
| Statins use | No | 5035 | 505 (10.0%) | 1.24 (1.01-1.52) | 0.037 | 0.550 |
|  | Yes | 869 | 93 (10.7%) | 1.72 (1.07-2.75) | 0.024 |  |

**Notes:**

**Adjustment:** Each subgroup model was adjusted for all covariates in the fully adjusted Model 3, excluding the stratification variable itself. **P for interaction:** Derived from likelihood ratio tests comparing models with and without the interaction term between CLR and the stratification variable.

**Abbreviations:** BMI, body mass index; CI, confidence interval; CLR, C-reactive protein-to-lymphocyte ratio; eGFR, estimated glomerular filtration rate.

**Supplemental Table S9. Analysis of Biological Interaction on the Additive Scale Between CLR and Blood Pressure Control.**

| Subgroup / Interaction Metric | HR (95% CI) for  High CLR (Q4) vs. Low CLR(Q1-Q3) | P value |
| --- | --- | --- |
| Blood pressure control |  |  |
| Controlled | 1.24 (0.86-1.78) | 0.255 |
| Uncontrolled | 1.37 (1.11-1.70) | 0.004 |
| Interaction Indices |  |  |
| Multiplicative P value | 0.445 |  |
| Additive Interaction (RERI) | 0.30 |  |
| Attributable Proportion (AP) | 0.17 |  |
| Synergy Index (SI) | 1.70 |  |

**Notes:**
**Model Adjustment:** The model was adjusted for all covariates in the fully adjusted Model 3, excluding systolic and diastolic blood pressure.

**Definitions:**

- **High CLR:** Defined as the highest quartile Q4 (CLR ≥ 1.815).
- **Uncontrolled BP:** Defined as SBP ≥ 140 mmHg and/or DBP ≥ 90 mmHg.

**Abbreviations:** CI, confidence interval; CKD, chronic kidney disease; CLR, C-reactive protein-to-lymphocyte ratio; HR, hazard ratio.

**Supplemental Table S10. Comparison of Predictive Performance Among Systemic Inflammatory Indices for Proteinuria-Onset CKD**

|  | Association Strength | | Discrimination | Reclassification (vs. Base Model) | | | |
| --- | --- | --- | --- | --- | --- | --- | --- |
| Index | Adjusted HR (95% CI) | P Value | 3-Year AUC | IDI | P Value | NRI | P Value |
| CLR | 1.14 (1.05-1.24) | 0.001 | 0.660 | 0.0040 | 0.008 | 0.083 | 0.138 |
| CAR | 1.12 (1.04-1.21) | 0.002 | 0.658 | 0.0030 | 0.007 | 0.098 | 0.139 |
| SIRI | 1.08 (0.99-1.17) | 0.074 | 0.655 | 0.0010 | 0.006 | 0.040 | 0.095 |
| AISI | 1.08 (0.99-1.17) | 0.090 | 0.655 | 0.0010 | 0.004 | 0.022 | 0.072 |
| SII | 1.05 (0.96-1.14) | 0.258 | 0.655 | 0.0006 | 0.003 | 0.021 | 0.066 |
| NLR | 1.04 (0.96-1.13) | 0.359 | 0.654 | 0.0005 | 0.003 | 0.039 | 0.093 |
| PLR | 1.01 (0.92-1.10) | 0.909 | 0.654 | <0.0001 | 0.001 | 0.040 | 0.081 |

**Notes:** Adjusted for all covariates in Model 3 (demographics, comorbidities, lifestyle, metabolic profiles, and medications), excluding any inflammatory markers. **Statistics:** Adjusted HR: Per 1-standard deviation (SD) increase in the log-transformed marker. IDI & NRI: Calculated at 3-year follow-up relative to the Base Model. P-values derived from 200 bootstrap iterations.

**Abbreviations:** AISI, Aggregate Index of Systemic Inflammation; AUC, Area Under the Curve; CAR, C-reactive Protein-to-Albumin Ratio; CLR, C-reactive Protein-to-Lymphocyte Ratio; IDI, Integrated Discrimination Improvement; NLR, Neutrophil-to-Lymphocyte Ratio; NRI, Net Reclassification Improvement; PLR, Platelet-to-Lymphocyte Ratio; SII, Systemic Immune-Inflammation Index; SIRI, Systemic Inflammation Response Index.

**Supplemental Table S11. Calibration of the CLR-Integrated Prediction Model by Risk Quartiles**

| Risk Group (Quartiles) | No. of Patients | Events | Mean Predicted Risk (%) | Observed Risk (KM %) | O/E Ratio |
| --- | --- | --- | --- | --- | --- |
| Low | 1,476 | 79 | 4.6% | 4.6% | 1.01 |
| Medium-Low | 1,476 | 116 | 7.2% | 7.8% | 1.08 |
| Medium-High | 1,476 | 159 | 10.2% | 10.0% | 0.98 |
| High | 1,476 | 244 | 17.6% | 17.2% | 0.98 |
| Note: O/E Ratio close to 1 indicates good calibration. <1 indicates overestimation, >1 indicates underestimation. | | | | | |

**Notes:** The table assesses the agreement between the predicted 3-year risk and the observed 3-year risk (Kaplan-Meier estimate) across quartiles of predicted risk. O/E Ratio (Observed/Expected Ratio): A ratio close to 1.0 indicates good calibration.

**Abbreviations:** CLR, C-reactive protein-to-lymphocyte ratio; KM, Kaplan-Meier.

**Supplemental Table S12. Risk Stratification Using the Optimal CLR Cutoff Value**

| CLR Group | Events / N (%) | Incidence Rate (per 1000 person-years) | Crude HR  (95% CI) | P value | Adjusted HR  (95% CI) | P value |
| --- | --- | --- | --- | --- | --- | --- |
| CLR < 1.37 | 336 / 3697 (9.1%) | 30.58 | 1.00 (Ref) |  | 1.00 (Ref) |  |
| CLR ≥ 1.37 | 262 / 2207 (11.9%) | 46.05 | 1.47 (1.25-1.72) | <0.001 | 1.41 (1.19-1.67) | <0.001 |

**Notes:**

**Optimal Cutoff (1.37):** Determined by maximizing the Youden Index (Sensitivity 43.8%, Specificity 63.3%) in the Receiver Operating Characteristic (ROC) analysis.

**Adjusted HR:** Derived from the fully adjusted Model 3, comparing participants with CLR ≥ 1.37 to those with CLR < 1.37.

**Consistency Across Ethnic Subgroups**: The predictive performance of the 1.37 cutoff remained consistent across different demographic backgrounds. In pre-specified subgroup analyses, the adjusted HRs for proteinuria-onset CKD (CLR ≥ 1.37 vs < 1.37) were 1.37 (95% CI 1.10-1.71) for the Han population, 1.55 (95% CI 1.09-2.22) for the Uyghur population, and 1.28 (95% CI 0.83-1.99) for other ethnicities. No significant modifying effect of ethnicity was observed (P for interaction = 0.514).

**Optimal Cutoff (1.37):** Determined by maximizing the Youden Index (Sensitivity + Specificity - 1) in the Receiver Operating Characteristic (ROC) analysis.

**Diagnostic Performance:** At this cutoff, the Sensitivity was 43.8% and Specificity was 63.3%.
**Adjusted HR:** Derived from the fully adjusted Model 3, comparing participants with CLR ≥ 1.37 to those with CLR < 1.37.
**Abbreviations:** CI, confidence interval; CLR, C-reactive protein-to-lymphocyte ratio; HR, hazard ratio.

**Supplemental Table S13. Sensitivity Analysis Excluding Participants with Events Occurring Within the First 3 Months of Follow-up**

| Exposure | Events/N | Crude Model HR (95% CI) P | Model 1 HR (95% CI) P | Model 2 HR (95% CI) P | Model 3 HR (95% CI) P |
| --- | --- | --- | --- | --- | --- |
| Per 1-SD increase | 453 / 4966 (9.1%) | 1.16 (1.06-1.27) <0.001 | 1.18 (1.08-1.29) <0.001 | 1.15 (1.05-1.26) 0.003 | 1.13 (1.03-1.24) 0.011 |
| Quartiles |  |  |  |  |  |
| Q1 (Lowest) | 109 / 1278 (8.5%) | 1.00 (Ref) | 1.00 (Ref) | 1.00 (Ref) | 1.00 (Ref) |
| Q2 | 107 / 1230 (8.7%) | 1.13 (0.87-1.48) 0.363 | 1.11 (0.85-1.46) 0.425 | 1.09 (0.83-1.42) 0.549 | 1.06 (0.81-1.39) 0.668 |
| Q3 | 108 / 1250 (8.6%) | 1.22 (0.93-1.59) 0.146 | 1.23 (0.94-1.61) 0.127 | 1.17 (0.89-1.54) 0.251 | 1.12 (0.85-1.47) 0.423 |
| Q4 (Highest) | 129 / 1208 (10.7%) | 1.53 (1.18-1.97) 0.001 | 1.60 (1.24-2.08) <0.001 | 1.47 (1.13-1.91) 0.004 | 1.38 (1.05-1.81) 0.019 |
| P for trend |  | 0.001 | <0.001 | 0.004 | 0.019 |
| High Risk Group |  |  |  |  |  |
| Q1-Q3 (Reference) | 324 / 3758 (8.6%) | 1.00 (Ref) | 1.00 (Ref) | 1.00 (Ref) | 1.00 (Ref) |
| Q4 (Highest) | 129 / 1208 (10.7%) | 1.38 (1.12-1.69) 0.002 | 1.44 (1.17-1.77) <0.001 | 1.35 (1.10-1.67) 0.005 | 1.30 (1.05-1.61) 0.015 |

**Note:** Adjusted for the same covariates as in the primary analysis (as detailed in Table 2).
**Abbreviations:** CI, confidence interval; HR, hazard ratio.

**Supplemental Table S14. Sensitivity Analysis Excluding Participants with Events Occurring Within the First 6 Months of Follow-up**

| Exposure | Events/N (%) | Crude Model HR (95% CI) P | Model 1 HR (95% CI) P | Model 2 HR (95% CI) P | Model 3 HR (95% CI) P |
| --- | --- | --- | --- | --- | --- |
| Per 1-SD increase | 424 / 4650 (9.1%) | 1.17 (1.07-1.28) <0.001 | 1.20 (1.09-1.31) <0.001 | 1.17 (1.06-1.28) 0.001 | 1.14 (1.03-1.25) 0.008 |
| Quartiles |  |  |  |  |  |
| Q1 (Lowest) | 102 / 1222 (8.3%) | 1.00 (Ref) | 1.00 (Ref) | 1.00 (Ref) | 1.00 (Ref) |
| Q2 | 100 / 1157 (8.6%) | 1.14 (0.86-1.50) 0.361 | 1.12 (0.85-1.48) 0.410 | 1.10 (0.83-1.45) 0.521 | 1.07 (0.81-1.41) 0.655 |
| Q3 | 99 / 1151 (8.6%) | 1.21 (0.92-1.59) 0.183 | 1.23 (0.93-1.63) 0.139 | 1.17 (0.89-1.56) 0.266 | 1.11 (0.83-1.47) 0.473 |
| Q4 (Highest) | 123 / 1120 (11.0%) | 1.58 (1.21-2.05) <0.001 | 1.67 (1.28-2.19) <0.001 | 1.54 (1.17-2.02) 0.002 | 1.43 (1.08-1.88) 0.012 |
| P for trend |  | <0.001 | <0.001 | 0.002 | 0.013 |
| High Risk Group |  |  |  |  |  |
| Q1-Q3 (Reference) | 301 / 3530 (8.5%) | 1.00 (Ref) | 1.00 (Ref) | 1.00 (Ref) | 1.00 (Ref) |
| Q4 (Highest) | 123 / 1120 (11.0%) | 1.43 (1.16-1.76) <0.001 | 1.50 (1.21-1.86) <0.001 | 1.41 (1.14-1.75) 0.002 | 1.35 (1.08-1.67) 0.008 |

**Note:** Adjusted for the same covariates as in the primary analysis (as detailed in Table 2).
**Abbreviations:** CI, confidence interval; HR, hazard ratio.

**Supplemental Table S15. Sensitivity Analysis Excluding Participants with Baseline CRP > 10 mg/L**

| Exposure | Events/N (%) | Crude Model HR (95% CI) P | Model 1 HR (95% CI) P | Model 2 HR (95% CI) P | Model 3 HR (95% CI) P |
| --- | --- | --- | --- | --- | --- |
| Per 1-SD increase | 570 / 5705 (10.0%) | 1.21 (1.10-1.32) <0.001 | 1.24 (1.13-1.36) <0.001 | 1.20 (1.09-1.32) <0.001 | 1.19 (1.08-1.31) <0.001 |
| Quartiles |  |  |  |  |  |
| Q1 (Lowest) | 133 / 1476 (9.0%) | 1.00 (Ref) | 1.00 (Ref) | 1.00 (Ref) | 1.00 (Ref) |
| Q2 | 149 / 1476 (10.1%) | 1.25 (0.99-1.58) 0.059 | 1.24 (0.98-1.57) 0.074 | 1.20 (0.95-1.52) 0.120 | 1.18 (0.93-1.50) 0.163 |
| Q3 | 142 / 1476 (9.6%) | 1.25 (0.99-1.59) 0.062 | 1.27 (1.00-1.61) 0.050 | 1.21 (0.95-1.54) 0.121 | 1.17 (0.92-1.50) 0.197 |
| Q4 (Highest) | 146 / 1277 (11.4%) | 1.59 (1.26-2.01) <0.001 | 1.68 (1.32-2.13) <0.001 | 1.56 (1.22-1.98) <0.001 | 1.50 (1.17-1.92) 0.001 |
| P for trend |  | <0.001 | <0.001 | <0.001 | 0.002 |
| High Risk Group |  |  |  |  |  |
| Q1-Q3 (Reference) | 424 / 4428 (9.6%) | 1.00 (Ref) | 1.00 (Ref) | 1.00 (Ref) | 1.00 (Ref) |
| Q4 (Highest) | 146 / 1277 (11.4%) | 1.37 (1.13-1.65) 0.001 | 1.44 (1.19-1.74) <0.001 | 1.37 (1.13-1.66) 0.002 | 1.34 (1.10-1.62) 0.004 |

**Note:** Adjusted for the same covariates as in the primary analysis (as detailed in Table 2).
**Abbreviations:** CI, confidence interval; CRP, C-reactive protein; HR, hazard ratio.

**Supplemental Table S16. Sensitivity Analysis Using Winsorization at the 99th Percentile**

| Exposure (Winsorized) | Events/N (%) | Crude Model HR (95% CI) P | Model 1 HR (95% CI) P | Model 2 HR (95% CI) P | Model 3 HR (95% CI) P |
| --- | --- | --- | --- | --- | --- |
| Per 1-SD increase | 598 / 5904 (10.1%) | 1.18 (1.09-1.27) <0.001 | 1.20 (1.11-1.30) <0.001 | 1.17 (1.08-1.27) <0.001 | 1.15 (1.06-1.24) 0.001 |
| Quartiles |  |  |  |  |  |
| Q1 (Lowest) | 133 / 1476 (9.0%) | 1.00 (Ref) | 1.00 (Ref) | 1.00 (Ref) | 1.00 (Ref) |
| Q2 | 149 / 1476 (10.1%) | 1.25 (0.99-1.58)  0.058 | 1.24 (0.98-1.56) 0.077 | 1.20 (0.95-1.52) 0.122 | 1.18 (0.93-1.49) 0.171 |
| Q3 | 142 / 1476 (9.6%) | 1.25 (0.99-1.59)  0.061 | 1.26 (0.99-1.60) 0.057 | 1.21 (0.95-1.53) 0.128 | 1.17 (0.92-1.49) 0.213 |
| Q4 (Highest) | 174 / 1476 (11.8%) | 1.60 (1.27-2.00) <0.001 | 1.67 (1.33-2.10) <0.001 | 1.54 (1.22-1.95) <0.001 | 1.46 (1.15-1.85) 0.002 |
| P for trend |  | <0.001 | <0.001 | <0.001 | 0.002 |
| High Risk Group |  |  |  |  |  |
| Q1-Q3 (Reference) | 424 / 4428 (9.6%) | 1.00 (Ref) | 1.00 (Ref) | 1.00 (Ref) | 1.00 (Ref) |
| Q4 (Highest) | 174 / 1476 (11.8%) | 1.37 (1.15-1.64) <0.001 | 1.44 (1.20-1.72) <0.001 | 1.36 (1.13-1.63) 0.001 | 1.31 (1.09-1.57) 0.004 |

**Note:** Adjusted for the same covariates as in the primary analysis (as detailed in Table 2).
**Abbreviations:** CI, confidence interval; HR, hazard ratio.

**Supplemental Table S17. Sensitivity Analysis with Administrative Censoring at 3 Years**

| Exposure | Events/N (%) | Crude Model HR (95% CI) P | Model 1 HR (95% CI) P | Model 2 HR (95% CI) P | Model 3 HR (95% CI) P |
| --- | --- | --- | --- | --- | --- |
| Per 1-SD increase | 420 / 5904 (7.1%) | 1.20 (1.09-1.31) <0.001 | 1.21 (1.11-1.33) <0.001 | 1.18 (1.07-1.30) <0.001 | 1.17 (1.06-1.29) 0.001 |
| Quartiles |  |  |  |  |  |
| Q1 (Lowest) | 86 / 1476 (  5.8%) | 1.00 (Ref) | 1.00 (Ref) | 1.00 (Ref) | 1.00 (Ref) |
| Q2 | 104 / 1476 (7.0%) | 1.31 (0.99-1.75)  0.062 | 1.29 (0.97-1.72) 0.081 | 1.26 (0.94-1.67) 0.119 | 1.24 (0.93-1.65) 0.142 |
| Q3 | 104 / 1476 (7.0%) | 1.35 (1.02-1.80)  0.039 | 1.34 (1.01-1.79) 0.046 | 1.30 (0.97-1.73) 0.078 | 1.28 (0.96-1.71) 0.098 |
| Q4 (Highest) | 126 / 1476 (8.5%) | 1.69 (1.29-2.23) <0.001 | 1.74 (1.32-2.30) <0.001 | 1.61 (1.21-2.14) 0.001 | 1.57 (1.18-2.09) 0.002 |
| P for trend |  | <0.001 | <0.001 | 0.001 | 0.002 |
| High Risk Group |  |  |  |  |  |
| Q1-Q3 (Reference) | 294 / 4428 (6.6%) | 1.00 (Ref) | 1.00 (Ref) | 1.00 (Ref) | 1.00 (Ref) |
| Q4 (Highest) | 126 / 1476 (8.5%) | 1.39 (1.13-1.72) 0.002 | 1.44 (1.17-1.78) <0.001 | 1.35 (1.09-1.68) 0.006 | 1.33 (1.07-1.65) 0.010 |

**Note:** Adjusted for the same covariates as in the primary analysis (as detailed in Table 2).
**Abbreviations:** CI, confidence interval; HR, hazard ratio.

**Supplemental Table S18. Sensitivity Analysis Excluding Current Smokers**

| Exposure | Events/N (%) | Crude Model HR (95% CI) P | Model 1 HR (95% CI) P | Model 2 HR (95% CI) P | Model 3 HR (95% CI) P |
| --- | --- | --- | --- | --- | --- |
| Per 1-SD increase | 383 / 4067 (9.4%) | 1.18 (1.08-1.30) <0.001 | 1.20 (1.09-1.33) <0.001 | 1.18 (1.07-1.31) <0.001 | 1.16 (1.05-1.28) 0.004 |
| Quartiles |  |  |  |  |  |
| Q1 (Lowest) | 83 / 1000 (8.3%) | 1.00 (Ref) | 1.00 (Ref) | 1.00 (Ref) | 1.00 (Ref) |
| Q2 | 94 / 1003 (9.4%) | 1.21 (0.90-1.63) 0.198 | 1.18 (0.88-1.59) 0.269 | 1.16 (0.86-1.57) 0.318 | 1.10 (0.82-1.49) 0.514 |
| Q3 | 88 / 1019 (8.6%) | 1.18 (0.88-1.60) 0.271 | 1.18 (0.87-1.59) 0.295 | 1.16 (0.86-1.58) 0.334 | 1.10 (0.81-1.50) 0.556 |
| Q4 (Highest) | 118 / 1045 (11.3%) | 1.66 (1.25-2.20) <0.001 | 1.72 (1.29-2.29) <0.001 | 1.65 (1.23-2.22) <0.001 | 1.53 (1.14-2.06) 0.005 |
| P for trend |  | <0.001 | <0.001 | 0.001 | 0.006 |
| High Risk Group |  |  |  |  |  |
| Q1-Q3 (Reference) | 265 / 3022 (8.8%) | 1.00 (Ref) | 1.00 (Ref) | 1.00 (Ref) | 1.00 (Ref) |
| Q4 (Highest) | 118 / 1045 (11.3%) | 1.47 (1.18-1.83) <0.001 | 1.54 (1.23-1.92) <0.001 | 1.49 (1.19-1.86) <0.001 | 1.43 (1.14-1.79) 0.002 |

**Note:** Adjusted for the same covariates as in the primary analysis (as detailed in Table 2).
**Abbreviations:** CI, confidence interval; HR, hazard ratio.

**Supplemental Table S19. Sensitivity Analysis Excluding Current Drinkers**

| Exposure | Events/N (%) | Crude Model HR (95% CI) P | Model 1 HR (95% CI) P | Model 2 HR (95% CI) P | Model 3 HR (95% CI) P |
| --- | --- | --- | --- | --- | --- |
| Per 1-SD increase | 388 / 4108 (9.4%) | 1.19 (1.09-1.31) <0.001 | 1.21 (1.10-1.33) <0.001 | 1.18 (1.07-1.30) 0.001 | 1.14 (1.03-1.26) 0.009 |
| Quartiles |  |  |  |  |  |
| Q1 (Lowest) | 86 / 1029 (8.4%) | 1.00 (Ref) | 1.00 (Ref) | 1.00 (Ref) | 1.00 (Ref) |
| Q2 | 93 / 995 (9.3%) | 1.25 (0.93-1.67) 0.138 | 1.24 (0.92-1.66) 0.159 | 1.18 (0.88-1.58) 0.277 | 1.11 (0.82-1.50) 0.491 |
| Q3 | 83 / 1003 (8.3%) | 1.13 (0.84-1.53) 0.424 | 1.13 (0.83-1.53) 0.432 | 1.08 (0.80-1.47) 0.612 | 0.99 (0.73-1.36) 0.967 |
| Q4 (Highest) | 126 / 1081 (11.7%) | 1.70 (1.29-2.24) <0.001 | 1.77 (1.33-2.34) <0.001 | 1.63 (1.22-2.17) <0.001 | 1.49 (1.11-1.99) 0.008 |
| P for trend |  | <0.001 | <0.001 | 0.002 | 0.013 |
| High Risk Group |  |  |  |  |  |
| Q1-Q3 (Reference) | 262 / 3027 (8.7%) | 1.00 (Ref) | 1.00 (Ref) | 1.00 (Ref) | 1.00 (Ref) |
| Q4 (Highest) | 126 / 1081 (11.7%) | 1.52 (1.23-1.88) <0.001 | 1.58 (1.27-1.96) <0.001 | 1.50 (1.20-1.87) <0.001 | 1.44 (1.15-1.80) 0.001 |

**Note:** Adjusted for the same covariates as in the primary analysis (as detailed in Table 2).
**Abbreviations:** CI, confidence interval; HR, hazard ratio.

**Supplemental Table S20. Sensitivity Analysis Excluding Participants with Diabetes Mellitus**

| Exposure | Events/N (%) | Crude Model HR (95% CI) P | Model 1 HR (95% CI) P | Model 2 HR (95% CI) P | Model 3 HR (95% CI) P |
| --- | --- | --- | --- | --- | --- |
| Per 1-SD increase | 547 / 5463 (10.0%) | 1.17 (1.08-1.27) <0.001 | 1.19 (1.09-1.29) <0.001 | 1.15 (1.06-1.25) <0.001 | 1.13 (1.04-1.23) 0.004 |
| Quartiles |  |  |  |  |  |
| Q1 (Lowest) | 122 / 1376 (8.9%) | 1.00 (Ref) | 1.00 (Ref) | 1.00 (Ref) | 1.00 (Ref) |
| Q2 | 137 / 1366 (10.0%) | 1.26 (0.99-1.61) 0.064 | 1.24 (0.97-1.58) 0.088 | 1.21 (0.94-1.54) 0.133 | 1.18 (0.92-1.51) 0.187 |
| Q3 | 133 / 1368 (9.7%) | 1.29 (1.01-1.65) 0.042 | 1.29 (1.01-1.65) 0.043 | 1.23 (0.96-1.58) 0.103 | 1.19 (0.93-1.54) 0.169 |
| Q4 (Highest) | 155 / 1353 (11.5%) | 1.58 (1.24-2.00) <0.001 | 1.63 (1.28-2.08) <0.001 | 1.50 (1.17-1.92) 0.001 | 1.43 (1.11-1.83) 0.005 |
| P for trend |  | <0.001 | <0.001 | 0.001 | 0.006 |
| High Risk Group |  |  |  |  |  |
| Q1-Q3 (Reference) | 392 / 4110 (9.5%) | 1.00 (Ref) | 1.00 (Ref) | 1.00 (Ref) | 1.00 (Ref) |
| Q4 (Highest) | 155 / 1353 (11.5%) | 1.34 (1.11-1.62) 0.002 | 1.39 (1.16-1.68) <0.001 | 1.31 (1.08-1.58) 0.006 | 1.26 (1.04-1.53) 0.018 |

**Notes:** Adjusted for the same covariates as in the primary analysis (as detailed in Table 2). Diabetes status was removed from the adjustment covariates in this model.

**Abbreviations:** CI, confidence interval; HR, hazard ratio.

**Supplemental Table S21. E-Values for the Association Between CLR and Proteinuria-Onset CKD**

| Exposure | Adjusted HR (95% CI) | E-value (Point Estimate) | E-value (Lower Confidence Limit) |
| --- | --- | --- | --- |
| Per 1-SD increase | 1.14 (1.05-1.24) | 1.54 | 1.29 |
| Q4 vs Q1 (Highest vs Lowest) | 1.46 (1.15-1.85) | 2.28 | 1.57 |
| High Risk Group (Q4 vs Q1-Q3) | 1.31 (1.09-1.57) | 1.94 | 1.40 |

**Note:** The E-value quantifies the minimum strength of association (on the risk ratio scale) that an unmeasured confounder would need to have with both the exposure (CLR) and the outcome (CKD) to explain away the observed exposure-outcome association, conditional on the measured covariates. Calculated based on the hazard ratios from the fully adjusted Model 3.
**Abbreviations:** CI, confidence interval; HR, hazard ratio.

**Supplemental Figure S1. Distribution of C-Reactive Protein-to-Lymphocyte Ratio (CLR) Before and After Logarithmic Transformation**


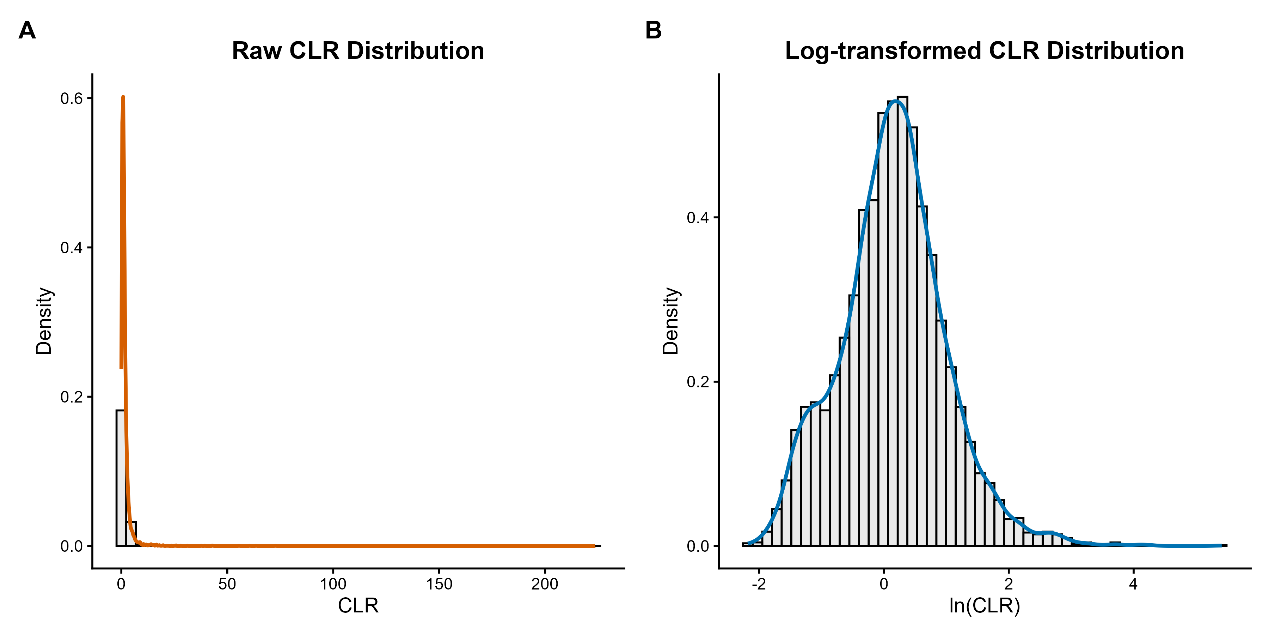


**(A) Raw CLR Distribution:** The histogram and density plot show a highly right-skewed distribution for the original CLR values.
**(B) ln-Transformed CLR Distribution:** After natural logarithmic transformation (ln-CLR), the distribution approximates normality.

**Supplemental Figure S2. Assessment of the Proportional Hazards Assumption for CLR**


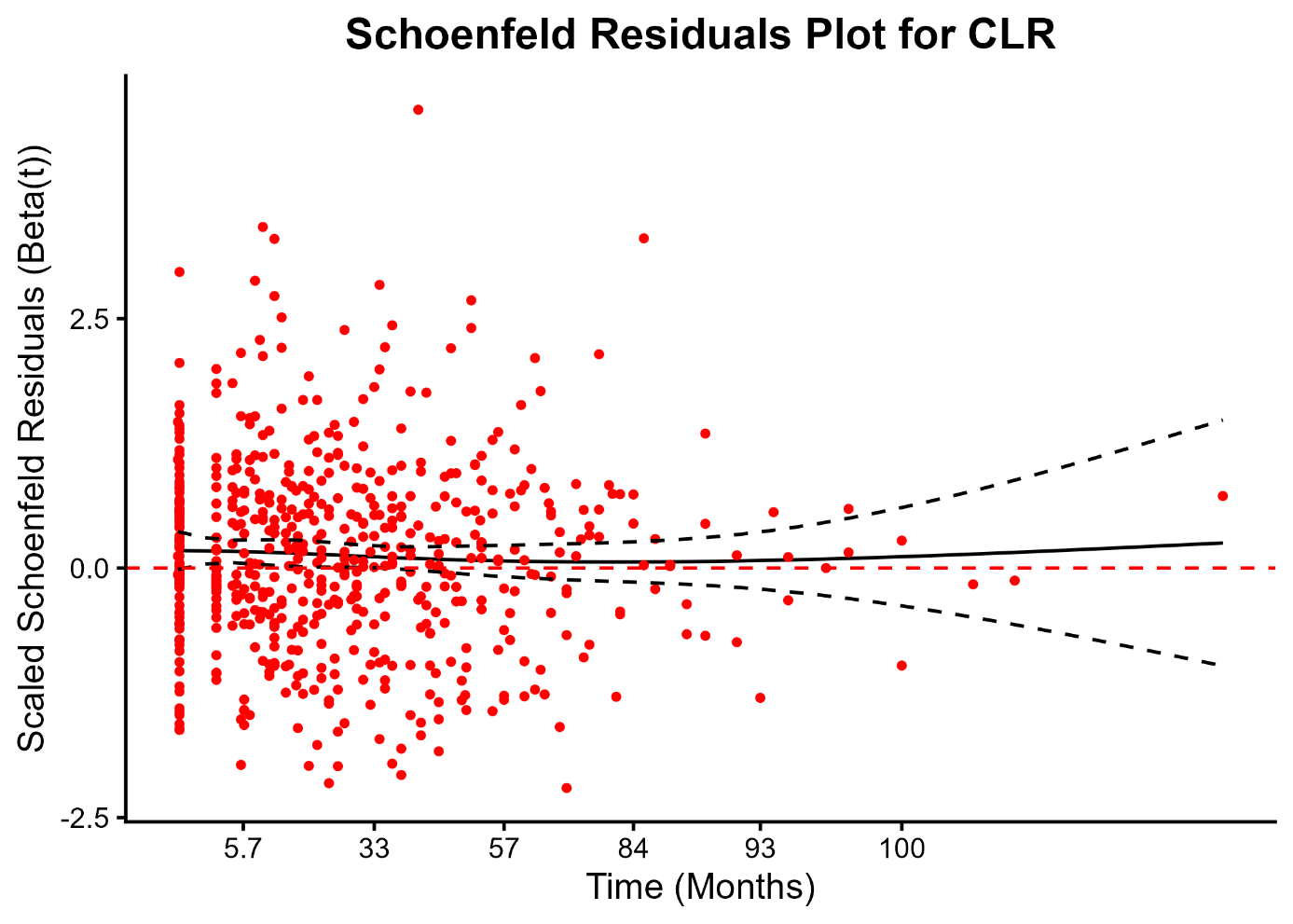


**Supplemental Figure S3. Cumulative Incidence of Proteinuria-Onset CKD Stratified by the Optimal CLR Cutoff**


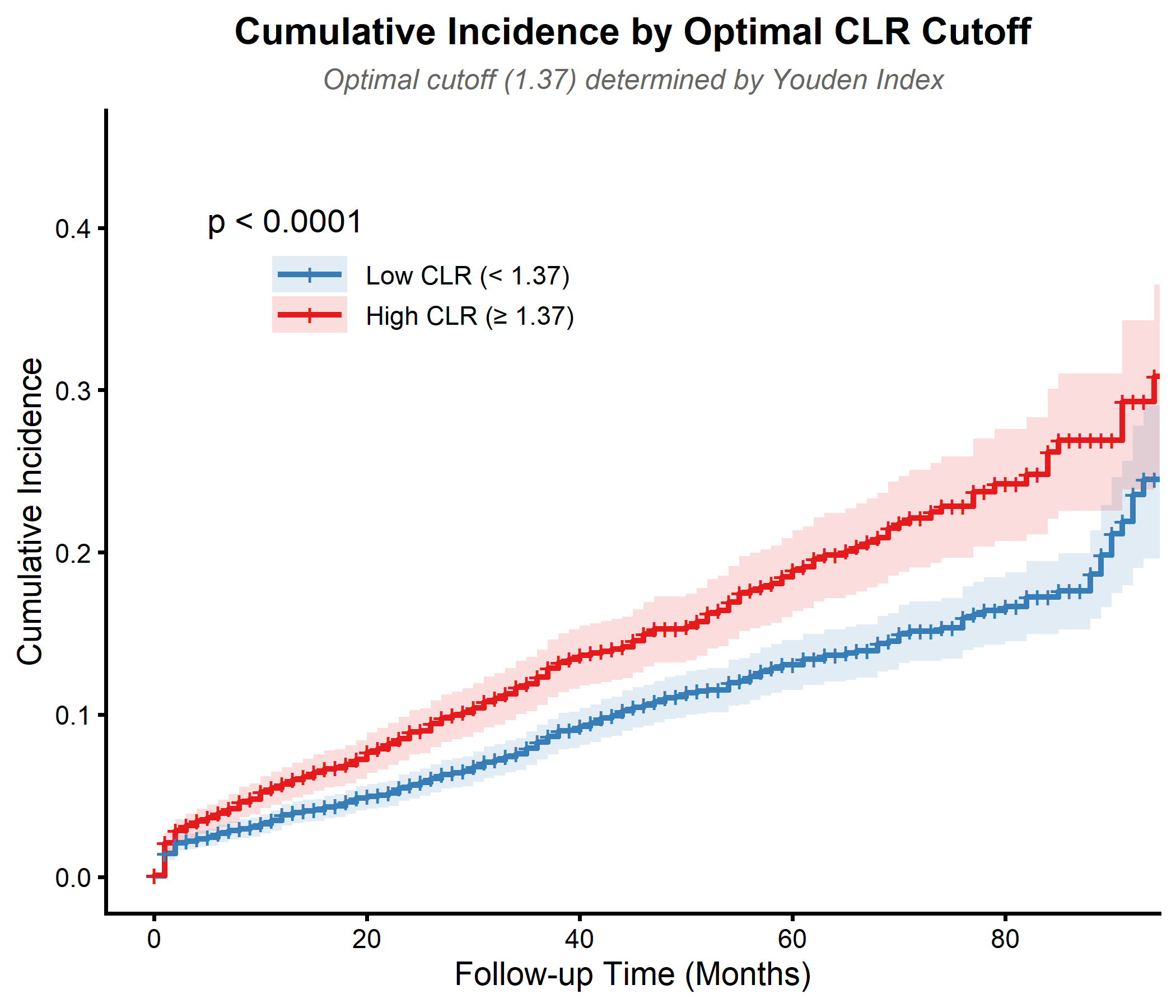

Supplement: Supplementary file 1 [file DataSheet1.docx]
